# Social mates dynamically coordinate aggressive behavior to produce strategic territorial defense

**DOI:** 10.1371/journal.pcbi.1012740

**Published:** 2025-01-24

**Authors:** Nicole M. Moody, Cole M. Williams, Sohini Ramachandran, Matthew J. Fuxjager

**Affiliations:** 1 Department of Ecology, Evolution, and Organismal Biology, Brown University, Providence, Rhode Island, United States of America; 2 Center for Computational Molecular Biology, Brown University, Providence, Rhode Island, United States of America; The University of Texas, UNITED STATES OF AMERICA

## Abstract

Negotiating social dynamics among allies and enemies is a complex problem that often requires individuals to tailor their behavioral approach to a specific situation based on environmental and/or social factors. One way to make these contextual adjustments is by arranging behavioral output into intentional patterns. Yet, few studies explore how behavioral patterns vary across a wide range of contexts, or how allies might interlace their behavior to produce a coordinated response. Here, we investigate the possibility that resident female and male downy woodpeckers guard their breeding territories from conspecific intruders by deploying defensive behavior in context-specific patterns. To study whether this is the case, we use correlation networks to reveal how suites of agonistic behavior are interrelated. We find that residents do organize their defense into definable patterns, with female and male social mates deploying their behaviors non-randomly in a correlated fashion. We then employ spectral clustering analyses to further distill these responses into distinct behavioral motifs. Our results show that this population of woodpeckers adjusts the defensive motifs deployed according to threat context. When we combine this approach with behavioral transition analyses, our results reveal that pair coordination is a common feature of territory defense in this species. However, if simulated intruders are less threatening, residents are more likely to defend solo, where only one bird deploys defensive behaviors. Overall, our study supports the hypothesis that nonhuman animals can pattern their behavior in a strategic and coordinated manner, while demonstrating the power of systems approaches for analyzing multiagent behavioral dynamics.

## Author summary

Understanding how individuals organize their behavior to accomplish specific goals is an ongoing question in animal behavior. One major challenge centers on measuring and comparing how different behavioral traits relate to each other. This becomes even more difficult when multiple individuals are working together toward a shared goal. Here, we investigate the complex dynamics by which animals organize defensive behaviors to protect territories. We find that social mates show signs of coordinating their activities, especially when it comes to fighting off strong intruders. We also find that population-level patterns of defense vary widely based on the type of challenger, with more threatening intruders triggering more complex defense from territorial residents. Our results demonstrate the unique insights available through a systems-based network approach to studying territoriality. We contribute to an emerging literature focused on using computational approaches to better illuminate the complex ways that animals can work in unison to tackle problems they face in the wild.

## Introduction

Strategizing is a major part of animal life. Work in humans elegantly demonstrates this point, with studies from preschool children [1] to corporate executives [2] showing that individuals dynamically adjust their behavior to optimally address novel situations and/or problems [3]. Yet, humans are not the only animals that appear to “strategize” in this manner—species from across the tree of life readily adopt different behavioral approaches when it comes to addressing challenges that include acquiring mates, finding food, and cooperating with allies [4–7]. However, despite this body of research, how animals organize their behavior into distinct patterns and adjust these behavioral patterns in response to contextual variation remains an open question.

Actively maintaining a territory is a facet of animal life where it is likely advantageous for individuals to intentionally choose how they pattern behavioral output. This is because territory residents should, in theory, act to defend their turf in a way that minimizes the costs of defense [8–10]. Theoretically, territory residents have a common goal to adjust their defense routines such that they are minimally sufficient at “fighting off” intruders [11–13]. What constitutes the minimum required behavioral effort to fight off an intruder likely varies based on numerous social and ecological factors [14–16], which can include the strength and/or quality of perceived territory challengers [17–19], level of resources within a territory [15,20], or even the physical layout of the territory itself [21]. All of these contextual factors, and others, may reasonably influence the behavioral patterns animals choose to deploy [22,23]. Further, in many cases throughout the animal world, territorial behavior is a multiagent endeavor, involving social allies who share access to resources [24–26]. Whether these allied individuals are social mates, kin, or neighbors, shared defense increases the potential complexity of the behavioral response. Allies, for example, might choose to work independently toward a shared goal of deterring intruders [27], or they may opt to more actively work together by coordinating their efforts to perform a shared defensive display [28,29]. While there are examples of such behavioral coordination in the literature, the details of how residents intertwine specific behaviors to generate coordinated patterns of defense remain understudied.

Here, we adopt a systems approach to quantify how wild animals arrange various behaviors over the course of a defensive bout. We take a population-wide look at how residents differentially deploy behaviors depending on the type of territorial threat they are facing, while also investigating how correlated multiagent efforts become a part of these defensive responses. We focus this work on free-living downy woodpeckers (*Dryobates pubescens*), a highly territorial species that is abundant across much of eastern North America [30,31]. Leading up to and during the breeding season, females and males form sociosexual partnerships, in which they establish and jointly defend the same breeding territory (<1 km^2^) [18]. These birds’ primary long-range territorial signal is the drum, a loud atonal acoustic display produced when individuals rapidly hammer their beak on hard substrates, such as trees or gutters [32–34]. Past work demonstrates that variation in a simulated territorial intruder’s drum length (number of beats) or drum speed (beats/sec; Hz) influences how resident woodpeckers defend their territory, with playback of higher performance in either category (i.e., drumming longer or faster) eliciting stronger defense responses from residents [18,19]. Further, there is evidence that social mates intentionally coordinate their behavior during defense, an approach that is perhaps even more common when intruders are perceived as especially threatening [18]. Notably, downy woodpeckers do not demonstrate any apparent sex-related differences in defensive behavior [18,32]. We therefore hypothesize that i) the deployment of defense behaviors follows distinct patterns ii) these behavioral patterns are tailored to the specific threat context and iii) residents often organize their territorial response with their social mate, coordinating their behavioral patterns over the course of a defensive bout.

Classically, most investigations of animal territorial behavior assess the level or magnitude of aggressive behavior [35–38]. However, a network-based computational approach to studying territorial interactions lets us view defensive behavior in a new light by showing the detailed patterns of how behaviors are produced relative to each other, and not just the frequency of behavior performance [39–41]. In other words, network methods allow us to characterize how behaviors are intertwined, which is relevant for understanding the context-specific tailoring we expect [4,7,18,26]. Here, we use correlation analyses to build behavioral networks that capture population-level patterns of defense by quantifying which behaviors are likely to co-occur, or not, over the duration of a territorial dispute.

To this end, we simulated different types of threatening intruders by broadcasting drums of different lengths and speeds to local woodpeckers, eliciting defensive behaviors from resident birds across variable threat contexts. We then used the residents’ behavioral responses in each threat context to create population-level correlational networks, which quantify both the positive and the negative relationships that exist between pairs of defense behavior. Next, we characterized behavioral motifs in each network using a computational method called spectral clustering, which separates suites of behavior in a way that maximizes within-motif similarity and among-motif dissimilarity [42]. Finally, we used transition analysis to interrogate the motifs for evidence of sequential pair coordination. Our use of spectral clustering is a novel application within the emerging field of network analyses for behavioral quantification.

## Results

### Initiation of territorial defense

In 92% of the simulated territorial intrusion playback sessions (58/63 sessions), we found that only one resident bird responded during our pre-stimulus whinny period (see Methods for details and justification of this design), and there was a trend for this individual to be a male rather than a female resident (*χ*^*2*^_1_ = 3.5, *p* = 0.06137). In many instances, a second bird was soon to join the first (43/63 sessions), with an average time delay between the first and second responder of 105 s (+/− 1SD = 160 s).

### Number of residents engaged in territorial defense

**Fig 1:**
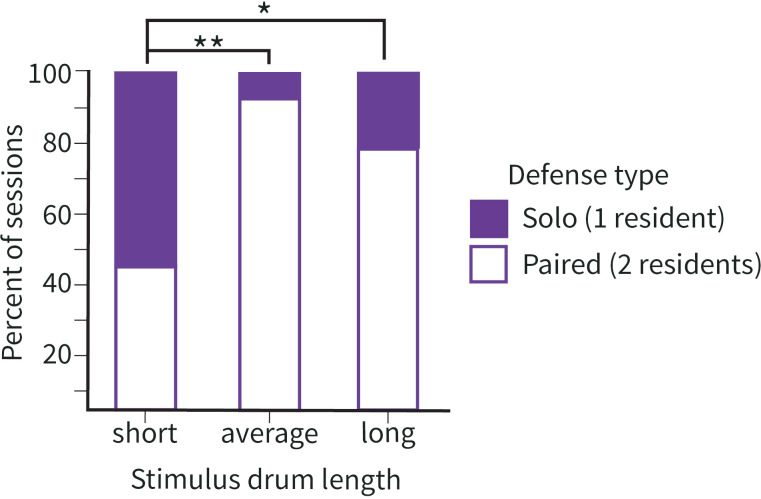
Drum length predicts number of residents involved in defense. Number of resident woodpeckers who engage in territorial defense varies based on the length of the simulated drum. Fisher’s exact test revealed that the proportion of solo responses varied based on drum length. Post-hoc tests showed that a resident is more likely to respond alone (solo defense) if the playback drum is short compared to both average length (** p < 0.01) and long (* p < 0.05) stimuli.

For 31% of simulated territorial intrusions (20/63 sessions), a second resident never joined the first responder. In these sessions, only one bird responded to the simulated intruder during the entire 10-min playback period, and thus we classified these defensive bouts as instances of “solo defense.” When we considered the threat context for these sessions, we see that “solo defense” was significantly more common in response to short drum stimuli (*p* < 0.01, two-tailed Fisher’s exact test; Fig 1). By contrast, when the playback drum length was either average or long, a second resident usually joined during the 10-min bout to create a “paired defense” response (43/63 sessions). Together, these results align with previous work in this species showing that longer drum lengths are perceived as a greater threat, and greater threats are more likely to be met by paired defense [18].

### Characterizing paired defense patterns in response to average drum stimuli

**Fig 2:**
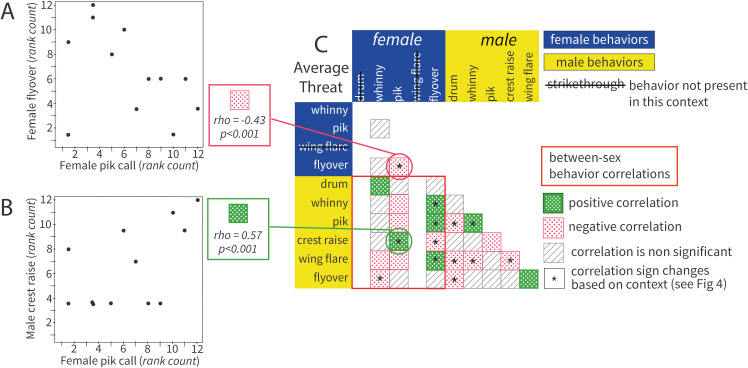
Behavioral correlations of population level paired response to an average threat simulated intruder. Population-wide correlations between defense behaviors performed during the 10-min defense bout in response to an average threat simulated intruder. **A)** Ranked behavior counts for an exemplar negative correlation. In this population and context, female flyovers and pik calls are generally not performed together in defense (rho = −0.43, p < 0.001). **B)** Ranked behavior counts for an exemplar positive correlation. In this population and context, female pik calls and male crest raises are generally performed together during a 10-min defense bout (rho = 0.57, p < 0.001). **C)** All colored squares indicate significant behavior correlations, where dotted green is a positive correlation and dotted red is a negative correlation. For exact correlation coefficients and p-values see supplement (S1 Table). Starred (*) boxes indicate a behavior pair correlation that is sometimes positive and sometimes negative, depending on the threat context (see Fig 4 for other contexts). Grey hatched squares represent non-significant correlations. Strikethrough behavior names indicate that behavior was not performed in response to an average simulated intruder. The orange box encompasses correlations that include behaviors performed by both the female and male resident.

We used correlation-based network analysis to quantify patterns of territorial behavior at the population level. As a first step in this process, we analyzed resident pair responses to the playback of average drum stimuli (see Methods) by calculating nonparametric, rank-based correlation coefficients (Spearman’s rho) between pairs of behavior deployed during the 10-min territorial intrusion (Fig 2A and 2B). This allowed us to establish how the performance of different behaviors related to each other in response to an average simulated intruder across the entire population.

Of the 38 possible behavioral correlations in the average threat context, we found that 20 were statistically significant compared to our null expectations (Fig 2C). Some of these behavioral correlations were positive, which means that across the population, resident birds tended to produce the behaviors together during a single 10-min simulated territorial intrusion (Fig 2B). Other behaviors were negatively correlated, indicating that resident birds in the population tended to perform those behaviors in isolation from each other (Fig 2A). In other words, if birds deployed a particular behavior, then they were less likely to also perform the negatively related behavior during that same 10-min timeframe. When correlation coefficients between two behaviors were not significantly different from our null expectation (see Methods: Generating a Null Expectation), then this indicated that one behavior’s performance was not predictive of whether the second behavior would occur during the 10-min defense bout.

**Fig 3:**
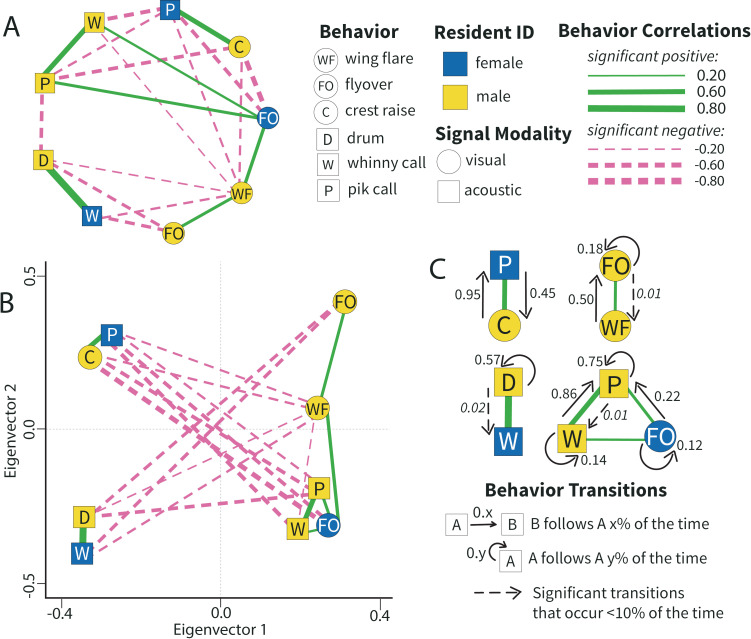
Behavioral network shows population level paired response to an average threat simulated intruder. Network analysis and clustering pipeline reveals distinct patterns in how resident pairs organize their defense. **A)** Territorial network graph where each shape (node) represents a behavior and the lines (edges) connecting nodes depict significant correlations. Solid green lines indicate positive correlations and dashed pink lines indicate negative correlations. Line width (edge weight) indicates the relative strength of the correlation (Spearman’s rho). **B)** Same territorial network plotted along eigenvector 1 (x-axis) and eigenvector 2 (y-axis) of the signed Laplacian matrix. Distance between nodes in eigenspace is informative, such that closer nodes are more similar in performance and more distant nodes are less similar. Nodes are jittered slightly for visualization. **C)** Motifs of related behaviors determined through spectral clustering of the full network. Each motif is composed of behaviors that are likely to be deployed together during a single territorial defense bout. Arrows indicate significant sequential transitions between behaviors, indicating that one behavior is likely to follow another x% of the time. Dashed arrows indicate that the transition likelihood was relatively low (<10%), but still significantly different than the null expectation. Distance in the motif panel is not informative.

Next, we used the correlation coefficients described above to build a population level network graph, where each node is a behavior and the edges (lines) connecting nodes represent statistically significant associations (Fig 3A). This basic network graph visualizes the direction (positive/negative) and magnitude of correlations between nodes. To visually represent the similarity and dissimilarity between behaviors’ performance, we plotted the network in the eigenspace of the graph’s signed Laplacian matrix (Fig 3B). The signed Laplacian matrix incorporates the strength and directionality of a node’s edges, as well as the node’s overall connectivity, such that the resulting position in eigenspace becomes quantitatively informative (see Methods: Quantifying Motifs for more details). In this final network visualization, behaviors (nodes) that are closer to each other were more similar in their performance than behaviors that are distant in eigenspace.

To quantify patterns that might emerge across the full response network, we used a technique called spectral clustering. This approach separated out motifs of positively related behaviors within the network using the eigenvectors of the graph’s signed Laplacian matrix. Importantly, with spectral clustering, motifs are clustered to simultaneously maximize both within-motif similarity and between-motif dissimilarity. As such, we found suites of positively related behavior that residents were more likely to co-produce when fighting off intruders in this population. In response to an average simulated intruder, we found four distinct motifs. Three of these motifs consisted of two positively correlated behaviors, whereas one motif consisted of three positively correlated behaviors (Fig 3C). Furthermore, three of the motifs included behaviors performed by both residents (i.e., between-sex motifs), and one motif was formed by only male behaviors. Given the largely negative edges between motifs in the summary response network, we can conclude that these four clusters of behavior were generally produced at the exclusion of each other. In other words, our results suggest that resident birds in this population were likely to deploy a particular motif in isolation from the other motifs that were negatively related to it.

Finally, to assess motif structure for patterns of sequential behavior deployment, we tested for significant transitions between positively correlated behaviors within motifs. We found that, at the population level, there are many behaviors whose performance is likely to follow a specific ordered pattern (Fig 3C). Some of these significant within-motif transitions were bidirectional, as is the case with female pik calls (blue P) and male crest raises (yellow C), whereas others were unidirectional as seen with female flyovers (blue FO) which were followed by male pik calls (yellow P) 22% of the time. Still, other transitions were self-referential, as seen with male drums (yellow D) that were produced in succession 57% of the time.

### Contextual differences in paired defense patterns

**Fig 4:**
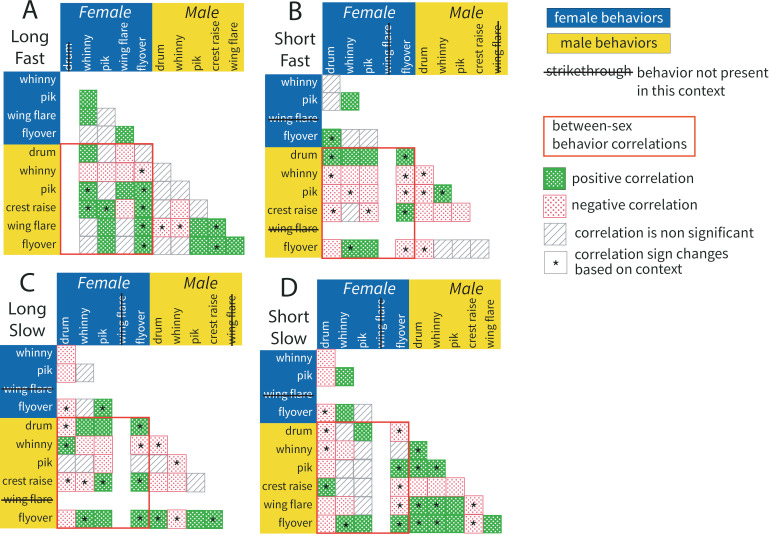
Behavioral correlations show contextual variation in paired defense. Patterns of behavioral correlations in paired territory defense vary across multiple threat contexts. All colored squares indicate significant behavioral correlations, where dotted green is a positive correlation and dotted red is a negative correlation. For exact Spearman’s rho correlation coefficients and p-values see supplement (S1 Table). Starred (*) boxes indicate a behavior pair correlation that is sometimes positive and sometimes negative, depending on the threat context. Grey hatched squares represent non-significant correlations. Strikethrough behavior names indicate the behavior was not performed in response to that simulated threat context. The orange box encompasses correlations that include behaviors performed by both the female and male resident. **A)** Population-level correlations for pair responses to simulated High Threat intruder (long/fast drum). **B)** Population-level correlations for pair responses to simulated Mixed Threat intruder (short/fast drum). **C)** Population-level correlations for pair responses to simulated Mixed Threat intruder (long/slow drum). **D)** Population-level correlations for pair responses to simulated Low Threat intruder (short/slow drum).

Next, we tested for population-level patterns in how resident pairs deploy defense behaviors in response to simulated intruders that represent different territorial threat levels. To do this we used the same analysis pipeline described above to create correlation-based behavioral networks, cluster suites of related behaviors into motifs, and analyze within-motif transitions. Accordingly, when we compared the population’s response to different types of simulated intruders, we saw context specific patterns in how behaviors were arranged for territory defense. Correlations between behaviors varied widely across contexts, with no pair of behaviors showing the same correlation across all threat contexts (Figs 2C and 4). Notably, some behaviors whose performance was positively related during defense in response to one type of simulated intruder were negatively correlated with each other in a different threat context. Such instances of significant correlations switching the directionality of the relationship are indicated in Figs 2C and 4 by asterisks (*).

**Fig 5:**
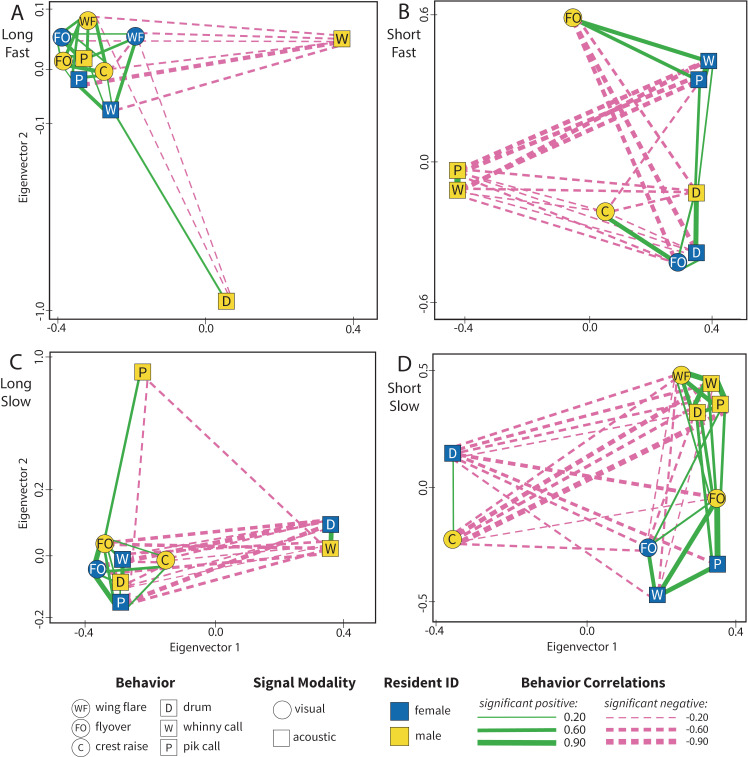
Paired territorial response networks vary across simulated threat contexts. Network graphs capture population-level behavioral patterns for paired defense in response to simulated territorial intruders. All edges (lines) indicate significant, non-random correlations between behaviors (nodes/shapes) where solid green is positive and dashed pink is negative and line width indicates the strength of the correlation (Spearman's rho). Paired defense networks are plotted along eigenvector 1 (x-axis) and eigenvector 2 (y-axis) of the signed Laplacian matrix and distance in these planes is informative such that closer nodes are more similar and more distant nodes are less similar. Nodes are jittered slightly for visualization. We have plotted each network on just two eigenvectors for readability. However, each of these paired defense networks was clustered using 3 or 4 eigenvectors for defining motifs (see Methods for more information and see S1 Fig for eigengap plots for each context). Population level networks for paired defense in response to simulated intruders of: **A)** High Threat (long/fast drum), **B)** Mixed Threat (short/fast drum), **C)** Mixed Threat (long/slow drum), and **D)** Low Threat (short/slow drum).

**Fig 6:**
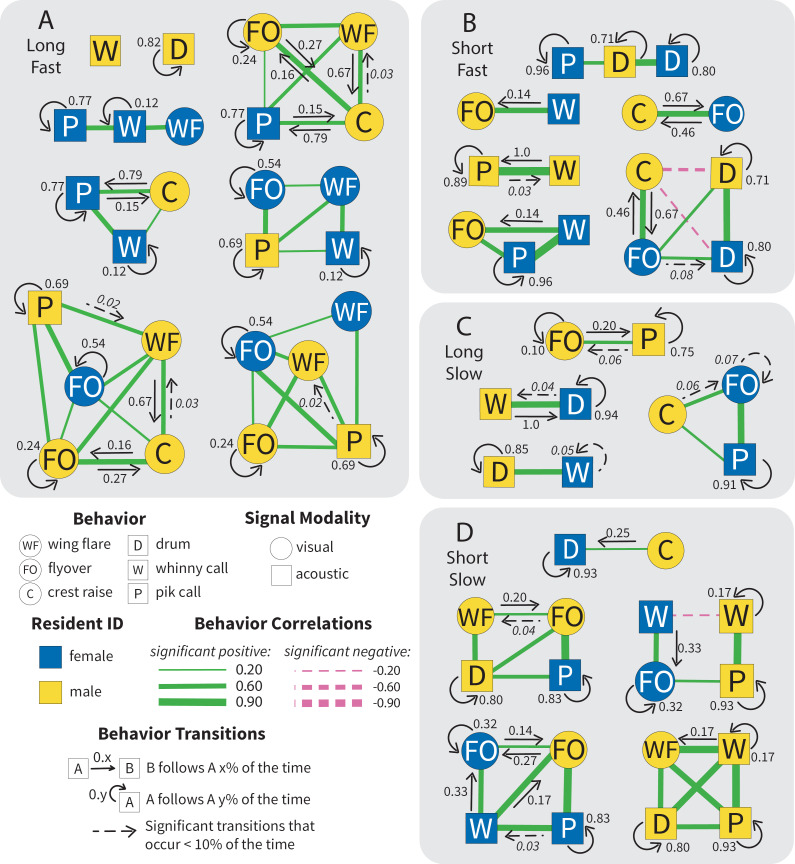
Paired territorial response networks cluster into diverse behavioral motifs. Within the larger network graph, motifs are subgraphs of mostly positively correlated behaviors, with few negative correlations within motifs (as determined by spectral clustering). Line width is indicative of the strength of the correlation between two behaviors; however, line length and relative position of nodes are NOT informative in this figure. Arrows show significant sequential transitions between two behaviors, with the number indicating the transitional probability. Dashed arrows are significant transitions that occur less than 10% of the time. Clustered motifs for population-wide paired defense responses to simulated intruders of: **A)** High Threat (long/fast drum), **B)** Mixed Threat (short/fast drum), **C)** Mixed Threat (long/slow drum), and **D)** Low Threat (short/slow drum).

**Fig 7:**
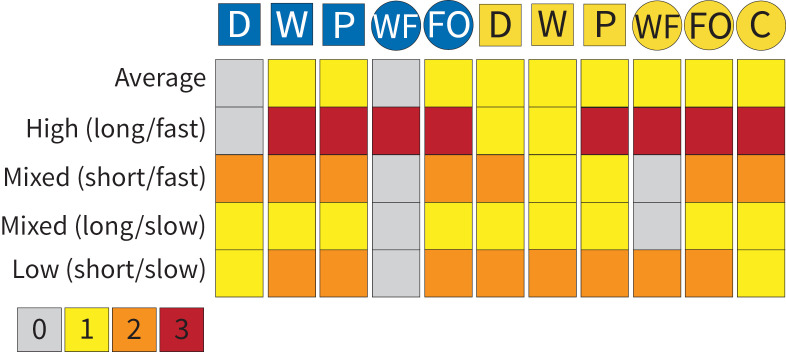
Behaviors can fill a role in multiple defense motifs. Heat map showing how many motifs each behavior clusters into within the population-level paired response networks. Behaviors that cluster into more than one motif may show functional variability within the broader defense landscape. The color corresponds to the number of motifs that a given behavior (column) clusters into for each threat context (row).

We next took all significant correlations and plotted them as network graphs in their signed Laplacian eigenspace (Fig 5). As above, this visualization put similar nodes close to each other and separated dissimilar nodes. In each context specific network, we saw that there are population-level patterns underlying how behaviors were deployed over a 10-min defense bout. Notably, the position of behavioral nodes relative to each other was unique for each threat context. Applying spectral clustering to the context specific network graphs allowed us to define motifs of co-occurring behaviors, as above (Fig 6). When comparing motifs across threat contexts, we found that the total number of motifs in a network varied based on the type of simulated intruder. Specifically, we saw only four motifs in response to the Long/Slow simulated intruder (Fig 6C), with three behavioral dyads and one behavioral triad, as found in the average threat response network (Fig 3C). By contrast, there were eight different motifs of positively related behaviors performed in response to the Long/Fast simulated intruder, and these motifs were composed of upwards of five different positively related behaviors (Fig 6A). Relatedly, as the number of motifs in a network increased, we saw that single behaviors were often present in more than one motif. The presence of a behavior in multiple motifs similarly varied as a function of the threat context, with response networks for high threat (Long/Fast) simulated intruders having the majority of behaviors (nodes) occupy positions in three distinct motifs (Fig 7).

Across all five contexts of paired defense, we found motifs with behaviors from both female and male residents, suggesting that between-sex correlations of behaviors are an important component of defense patterns across the population. Though, importantly, the behaviors from each sex that were positively correlated and found in between-sex motifs varied widely based on the type of threat. Similarly, across all threat contexts there were many significant transitions between female and male behaviors, indicating that residents were often performing their behaviors in sequence with their mate (Figs 3C and 6). Together, these results may suggest that residents adjusted how they linked up different behavioral traits during a defensive bout, and these adjustments occurred in response to the perceived threat posed by a simulated intruder.

### Patterns of solo defense

**Fig 8:**
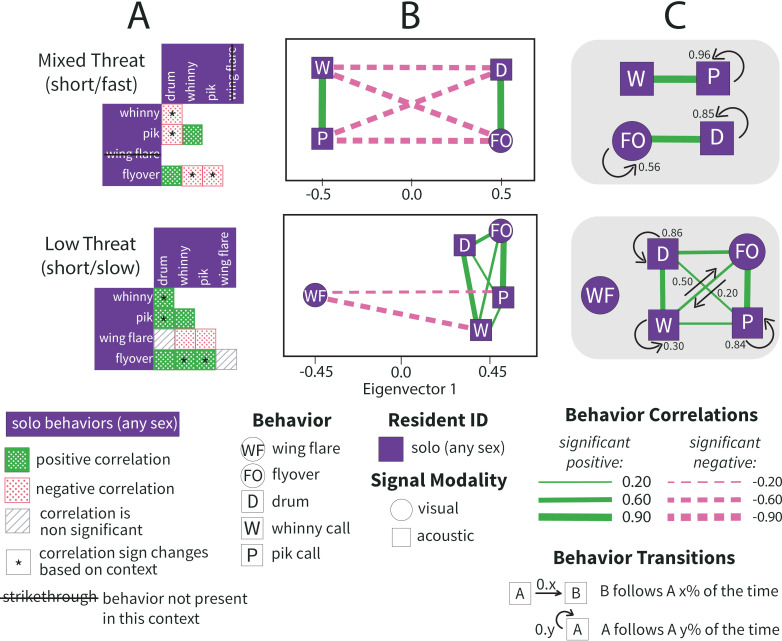
Population level solo responses to short drum stimuli. Population-wide correlations between defense behaviors performed during 10-min defense bout in response to short drum stimuli reveal distinct patterns in how residents organize solo defense. The top row of panels is solo response to Mixed Threat (short/fast) stimuli, the bottom row is solo response to Low Threat (short/slow) stimuli. **A)** All colored squares indicate significant behavior correlations, where dotted green is a positive correlation and dotted red is a negative correlation. For exact correlation coefficients and p-values see supplement (S1 Table). Starred (*) boxes indicate a behavior pair correlation that is sometimes positive and sometimes negative, depending on the threat context. Grey hatched squares represent non-significant correlations. Strikethrough behavior names indicate the behavior was not performed in that context. **B)** Solo defense network graphs capture population-level behavioral patterns. All edges (lines) indicate significant, non-random correlations between behaviors (nodes/shapes) where solid green is positive and dashed pink is negative, and line width indicates the strength of the correlation. Solo networks are plotted on eigenvector 1 (x-axis) of the signed Laplacian matrix because they were only clustered on eigenvector 1. Distance between nodes is informative only in the horizontal (x) direction, position in the vertical (y) direction is solely for visualization. Nodes are purple because these represent female and male responders. Nodes are jittered slightly for visualization. **C)** Motifs of related behaviors determined through spectral clustering of the full network. Each motif is composed of behaviors that are likely to be deployed together during a single territorial defense bout. Arrows represent significant sequential transitions between behaviors, indicating that one behavior is likely to follow another x% of the time. Distance in the motif panel is not informative.

As described above, solo defense was more likely to occur in response to playback of short drums than playback of average or long length drums (Fig 1). As such, we were only able to calculate solo behavioral defense networks for the Short/Slow and Short/Fast playback contexts. Each of these two playback contexts showed distinct patterns of significant behavioral correlations (Fig 8A). Both networks were plotted (Fig 8B) and clustered on only the first eigenvector of the signed Laplacian matrix. These population-level behavioral networks for solo defense each had two clustered motifs (Fig 8C). However, the structures of these motifs were quite different, with the large four behavior motif found in response to Short/Slow playback splitting into two dyadic motifs when the drum speed was greater during Short/Fast playback. In other words, when drum length was held constant (short), an increased threat context (faster intruding drum) changed the correlative relationships between behaviors, such that residents largely responded with dyadic motifs of behavior.

### Occurrence of behaviors across contexts

In a final analysis, we verified that there were no major differences in the frequency of behavior performance across threat contexts. Analyzing performance frequency is a classic approach in studies of territorial behavior [35–38]. In our study, the deployment of some behaviors did appear to be context specific (Fig 4). Specifically, performance of both male flyovers (Kruskal-Wallis *χ*^*2*^_4_ = 9.8512, *p* = 0.043) and male wing flares (Kruskal-Wallis *χ*^*2*^_4_ = 11.807, *p =* 0.019) varied as a function of playback type. Post-hoc analyses, however, showed no clear differences between groups (S2 Table). We found that all other behaviors were produced at statistically indistinguishable levels across treatments (S2 Table). While this finding of relatively consistent behavior frequencies may be surprising considering the past literature that focuses on performance frequency, these results underscore the need for novel approaches for quantifying patterns of territorial behavior.

## Discussion

Defending a territory is an important part of life for many animals. In downy woodpeckers, we find that territorial defense is a largely shared endeavor, in which socially mated female and male residents organize a collective behavioral response to simulated territory challengers. At the population level, paired defense networks are made up of multiple behavioral motifs, or modular suites of agonistic behavior, which are generated by both residents and often include between-sex sequences of behavior. We identified these motifs of related behavior through a novel application of spectral clustering. We find that the structure and deployment of our clustered motifs depends largely on an interaction’s threat context, suggesting that motif arrangement is tactical in nature and conditionally tailored to a particular defensive situation. Specifically, defense in response to high threat intruders is characterized by a greater number of behavioral clusters, with individual behaviors occupying roles in more motifs when compared to low threat intruders. Further, residents that engage in lower threat simulated encounters are more likely to deploy less behaviorally complex solo defense. Altogether, our findings suggest that territorial defense is a dynamic, context-dependent process, in which residents modulate how they intertwine and coordinate behaviors that underlie the expulsion of intruders.

### Quantifiable behavior patterns suggest behavioral strategy

Our results suggest that downy woodpeckers arrange their defensive behaviors in distinct patterns, captured through correlational analyses, which birds appear to adjust in response to changes in the severity of a territorial threat. In this regard, we find that in our population, resident social mates tend to produce specific combinations of behavior when they fight off different types of simulated intruders. We hypothesize that such responses reflect a certain degree of behavioral strategizing, akin to what we see in previous studies that denote “strategy” as the differential use of behavioral traits based on the context or condition of a given encounter [43–45].

One way to conceptualize the idea of strategic defense is by considering the null expectation, which is no strategizing. In such cases, we might expect that population-level correlations between behavioral traits would be indistinguishable from our simulated random expectations, resulting in statistically nonsignificant correlation coefficient values. This result would point to an absence of clear territorial behavior patterns, at least at the level of the population. Therefore, without discernable patterns of behavior deployment, we could not conclude that residents are broadly strategizing in their defense. Alternatively, a case of no strategizing could include some evidence of significant (non-random) behavioral correlations, but without any sign of these correlations differing across contexts. In theory, this would not qualify as conditional strategy *per se*, because individuals would not be changing how they pattern their behavior as a function of encounter context or condition [43,45]. Such seemingly fixed correlated suites of behavior might be more reminiscent of a behavioral syndrome [46], where individuals exhibit relatively less flexibility in their behavioral traits across contexts [47,48]. Instead, the observed differential use of behavioral traits based on some conditional factor—in our case, threat context—is likely indicative of strategic choice [22,23,43–45].

Here, we use emerging network methods to quantify how diverse behaviors are strategically deployed in a correlated fashion to accomplish territory defense. To date, most work on behavioral strategies has focused on reproductive and courtship behaviors [22,23,44,49,50]. While there is some discussion of strategic territoriality in the literature, previous research in this vein has largely focused on relatively simplistic definitions of strategy. These studies are usually limited to considering only one or two defense behaviors [51–53]. Though this is a helpful first step to demystifying territorial strategies, the reality of defense for many species is often much more complex, with defenders using myriad aggressive signals over the course of an encounter [12,52,54]. Given the wide range of challengers that may threaten a resident’s territory ownership, strategic use of the full range of available defense behaviors would seem to be highly advantageous from a fitness perspective [14,18,35,55]. Our approach allows us to uncover precisely how resident birds might deliberately stitch together many different behavioral traits when defending their home turf, and how these patterns might change in response to contextual variation. In our population, we found both positive and negative behavioral correlations, implying that there is likely functional value in both joining and separating the performance of certain behaviors during a territorial dispute. Further, the precise behaviors that are correlated with each other, and the directionality of those correlations, can vary widely across threat contexts in this system. Our findings are consistent with past work showing that diverse organisms intentionally arrange suites of behavior to address an array of complex “problems” such as courtship [56,57] and agonistic interactions [58,59].

From a methodological perspective, our study is novel in its use of spectral clustering to analyze behavioral networks. This approach clusters nodes within a network by incorporating information about both the directionality (sign) and the strength (weight) of edges (correlations). This provides a robust clustering of suites of similar behaviors whose performance was tightly correlated, while separating them from dissimilar behaviors. At the same time, spectral clustering offers several practical advantages for behavioral studies. It is fast, easy to implement, and does not require the pre-training needed by clustering algorithms based on neural networks. The method is parameter-free, such that you do not need to input a pre-determined number of clusters for the network to split into. Further, nodes can be present in more than one cluster (motif), which is biologically appropriate and relevant in behavioral contexts, particularly when studying communication signals that are often produced in variable combinations [39–41,60]. However, there are also considerations that researchers should keep in mind, including the impacts of sample size and rare behaviors. Here, the generalizability of some of our behavioral correlations is tenuous, specifically in our threat contexts with smaller sample sizes. For example, in paired response to a Short/Fast simulated intruder (N = 5), only one resident pair deployed the male crest raise (yellow C) behavior. Therefore, all correlations relating to this node are based on one defensive bout, and it would not be appropriate to draw functional conclusions about this relatively rare defense behavior from a small sample. As such, we offer our results as evidence that contextual differences exist, and there is still much work needed to understand the nuances of how particular downy woodpecker behaviors function in defense networks across all possible threat contexts. Here, we demonstrate the utility of clustering methods for hypothesis generation and highlight spectral clustering as one potentially useful approach.

### Behavioral motifs as a way to elucidate defensive tactics

If woodpecker territory defense is a strategic endeavor, then there should exist “tactics” of behavior that residents can choose from during a defensive bout. This is a key feature of strategy across taxa, where organisms have access to many alternative tactics and intentionally choose among them based on conditional or contextual factors [23,44,45]. Here, we hypothesize that tactics are reflected through the behavioral motifs that our spectral clustering approach uncovers. As described above, motifs are combinations of behavior that, across the population, are generally performed together during defense. Accordingly, our results suggest that motif composition is context-dependent, considering that the correlated behaviors which cluster into motifs vary depending on the type of simulated challenger. We propose that this indicates that territory defense involves the strategic deployment of behavioral tactics (motifs), and that residents make tactical choices based (at least in part) on the threat level of the intruder.

A tactical approach to territoriality would imply that, at the population level, residents choose among a variety of available behavioral tactics. In theory, this suggests that there can exist more tactics than any one individual or pair would likely use during a single defensive bout [43,45,61]. It is therefore not surprising that we find variation in the number of tactics present in the population’s response to different simulated intruders. Specifically, the response network elicited by our highest threat simulated intruder is composed of the most unique tactics (eight), whereas the average threat response network only contains four unique tactics. The number of tactics found in the population-level network could indicate how much variation there is among residents’ strategic decision making. Take, for example, the response network for average threat intruders. The population level network clusters into four clearly defined tactics with each behavior node positioned within a single tactic (Fig 3C). This may suggest that there is some population-wide consistency about how to best approach an average intruder, and resident pairs tactically choose to defend using one (or more) of these four relatively simple motifs. By contrast, the highest simulated threat context elicited a population-level network with eight unique motifs, where most behaviors are found in three distinct tactics. This implies that there is a larger “menu of tactics” for residents to choose from when they construct defense in a high threat context, and therefore perhaps greater variation between individual-level defense patterns. While we assert that threat context is an important determining factor for tactical structure, we cannot determine how residents choose between tactics in a given threat context in our current study. Though, the level of cognition required for residents to make informed decisions about tactic deployment is well within the realm of possibility [23,49,62]. Indeed, it is established that many species show evidence of decision making over the course of an interaction, whereby they use contextual information to choose what action they will take next [23,51,63,64].

Another important implication of our study is that combining and re-combining the same behaviors into different arrangements likely has some relevance for territory defense. This idea aligns with many other animal systems, in which combinations of behavior functionally represent something greater than the sum of their parts [60,65,66]. In this same way, we suspect that tactics (motifs) reflect behavioral configurations that have a functional meaning that is greater (or different) than any of the constitutive behaviors produced in isolation. For example, while pik calls have been hypothesized to function in pair coordination [18], it is possible that piks take on a different functional role when produced alongside whinny calls or a raised crest. We see that piks are often accompanied by these two behaviors in various threat contexts (Figs 3C and 6), possibly indicating that pik calls from the female inform a male’s crest raise. Or perhaps some threats are sufficiently addressed by an acoustic defense (i.e., whinny and pik combined) and don’t need an additional visual signal (crest raise). Of course, it is important to emphasize that our functional interpretations of tactics are purely speculation. While our results reveal the intricate behavioral structure of defensive tactics, our findings do not elucidate their functional meaning. We encourage future work designed to test the functional relevance of these behavioral motifs in downy woodpecker territoriality, as their structural differences may correspond to functional differences.

It will also be particularly important to study the prospect of strategic use of behavioral tactics at the level of individual territories. Our study was designed to capture and describe behavioral variation at the population level, and at this scale we do see evidence for the existence of strategy. However, to fully determine that strategic choice is at work in this system, we need to understand how patterns of behavior vary at the level of individual residents and pairs. For example, if residents make strategic choices about tactic deployment based on threat context as we propose here, we expect that pairs would switch which tactics they use in response to different playback stimuli. This question requires an experimental approach where focal territories are tested with playback stimuli repeatedly, and responses from each individual territory are compared between threat contexts. Additionally, beyond threat context, there are numerous other factors that could affect tactical choices at the level of individual territories such as individual or pair qualities [44,67], local population density [68], or qualities of the territory [20,69]. Further, spatial factors may influence aspects of strategic choice, such that certain tactics or behaviors generally occur in close proximity to either the (simulated) intruder or social mate [4]. Indeed, spatial proximity is important for tactical choice in male ostracods, who choose mating tactics based on their proximity to other males [23]. Or perhaps certain tactics are more likely to occur at the beginning or end of a simulated territorial intrusion, as seen in spiders, chiffchaffs, and bushcrickets [22,70,71]. We recognize that our point of view about territorial strategy and tactical structure may be provocative, and it certainly merits further investigation. Indeed, we suspect that the behavior patterns underlying defense can, and likely do, vary in many dimensions that we do not capture here. Our study is simply a first attempt at understanding how a population of woodpeckers might deliberately stitch together different behavioral traits when defending their home turf, and there is ample opportunity for future work.

### Territorial defense is often a coordinated effort

Coordination is often a mysterious process, in that we know animals routinely work together to accomplish a variety of tasks, but we seldom understand how they orchestrate their actions to do so. Behavioral coordination can occur when multiple individuals organize their activities to help accomplish a common goal [72–74]. In this study, resident pairs share a common goal of expelling a (simulated) conspecific intruder from their breeding territory. We find compelling evidence for coordination between pairs in the context specificity of a paired defensive approach, abundant between-sex behavior correlations, and sequential transitions between female and male behaviors.

First, we show evidence that a paired approach to territory defense may itself be a strategic choice in our system. Specifically, resident pairs of woodpeckers are more likely to respond together, coordinating their agonistic behaviors, when faced with a greater territorial threat (Fig 1). Such contextualization of coordination is consistent with our broader understanding of multiagent strategizing. Indeed, less challenging situations can frequently be solved by an individual adopting their own solo strategy, such as when humans are tasked with containing a small number of virtual sheep [75] or when an intruding woodpecker uses short drums in their challenge. However, in more challenging situations, a paired approach is often more effective. For example, virtually herding a large number of sheep is more successful when participants adopt a coordinated strategy and work together to contain the errant sheep [75]. Similarly, average length and longer drums tend to be met with coordinated, paired defense from resident woodpeckers. By deploying paired strategies when the challenge is greater, downy woodpeckers appear to apply this common principle of coordination to appropriately meet the behavioral challenge at hand.

Across all contexts, we found many significant between-sex behavior correlations in paired defense. We propose that the abundance of correlations between female and male behaviors at the population level is evidence of pairs coordinating their efforts to jointly defend a shared territory. This interpretation is consistent with defense in ovenbirds, where between-sex correlations of defensive behavior indicate residents coordinating their efforts in response to higher threat simulated intruders [72]. Some of the most iconic research on behavioral coordination looks at duetting birds, where the correlated performance of vocalizations by a social pair is understood as a mechanism by which residents can enhance the strength of their territorial display [74,76,77] and such coordination is associated with more territorial species [78,79]. We suspect that coordination of diverse territorial behaviors can function in a similar fashion to duetting. That is, female and male downy woodpeckers intentionally coordinate the performance of their agonistic displays, including various vocalizations, gestures, and other forms of movement (e.g., flyovers) to enhance their defense.

We see further evidence for direct, temporally based coordination in the significant transitions between behaviors performed by different sex residents. The sequential trading of behaviors between females and males supports the notion that one resident’s behavior can directly influence the behavior of their social mate. These between-individual behavioral sequences are consistent with evidence of coordination in other contexts, such as inter-species negotiations [80]. It is important to bear in mind, however, that a direct sequence of behaviors is not the only way residents can coordinate their activities. For example, in many sentinel species, a single individual (the sentinel) will keep watch for predators while the rest of the group forages [81–83]. This sentinel will consistently signal, vocalizing repeatedly to let their groupmates know that they are keeping watch. These vocalizations do not result in a direct behavioral change from the foraging individuals who continue their foraging, but instead increase the overall spread and ease of the group [81]. While this has been shown to be a coordinated activity among group living animals, a sequential analysis of this signaling would likely show transitions only within the sentinel’s own behavior. Similarly, in our woodpecker system, it is possible that one resident’s behavior could affect their mate’s vigilance, attention, and/or level of engagement with the intruding challenger. For example, pik calls have been hypothesized to serve an alert function between residents [18,84]. Here, we find high rates of transitions within one individual producing pik calls, indicated by pik nodes showing self-referential transitions of 69% or greater in all paired defense networks (Figs 3C and 6). In other words, in paired defense across all contexts, at least one resident tends to produce a sequence of piks. This repetitive signaling from one individual could reasonably affect how their mate is attending to the intruder, even if the mate does not perform a specific defense behavior during the series of piks. As such, significant between-sex behavioral transitions are only one way to capture pair coordination. Future work is needed to fully explore the functional meaning of the specific behavioral sequences that our work has begun to identify.

Similar to above, there are limitations to the interpretability of our transition analysis results. Specifically, infrequent behaviors and small sample sizes may bias some of the findings. For example, paired defense in a Long/Slow threat context shows a 100% transition probability between male whinny calls (yellow W) and female drums (blue D) (Fig 6C). This transition is statistically significant, though male whinny calls only occurred during one Long/Slow playback session. It would therefore be misguided to conclude that across the population, all resident pairs follow this sequential pattern. As such, we do not claim to reveal the specifics of temporal patterns of defense behavior. Instead, we aim to highlight the need for future research to explore how particular sequences are consistent or variable across territories and contexts. As above, an experiment designed to test behavioral strategizing and tactical choice at the individual territory level would be a natural next step for this work. Here, we demonstrate that there are numerous between-sex sequences of behavior across all threat contexts which, considered alongside our other lines of evidence, supports the idea that resident pairs coordinate their defense.

### Conclusions

Our findings suggest that territory defense in downy woodpeckers is a shared effort that often involves both members of a resident pair. We use networks to quantify patterns of behavior that persist at the population level and demonstrate context dependence, suggesting that territory defense is strategic in this system. We apply spectral clustering to our behavioral networks, revealing tactics (motifs) - suites of agonistic behavior whose performance is generally correlated across the population. Overall, the highest threat context elicited the most unique tactics in paired territorial defense, and lower threat simulated intruders were more likely to be met with solo defense. Altogether, we outline a framework for studying defensive strategy that aims to describe the complex dynamics involved in territorial signaling systems.

## Methods

### Ethics statement

All methods were approved by the Brown University Institutional Animal Care and Use Committee, the Rhode Island Department of Environmental Management, and the United States Fish and Wildlife Service.

### Animals

We studied territorial behavior in free-living downy woodpeckers in parks and greenspaces throughout Rhode Island, USA. Our field experiment took place during the birds’ breeding season (March – May 2021), which corresponds with the species’ main periods of territory establishment and defense prior to nest incubation [30]. Experimental sites were all at least 1 km apart to ensure that we did not perform playback on the same territory more than once. In this way, we were able to ensure that we only collected data from a resident bird or pair one time [18,19]. This approach is standard in studies of territoriality in birds [85–87].

### Simulated territorial intrusions

We used a classic simulated territorial intrusion experimental design, which consisted of broadcasting drum stimuli (see below) to resident birds and recording their responses during stimuli playback [for woodpeckers: 18, 19]. Once resident birds were located at a given site either by visually seeing the bird or hearing their characteristic drumming or vocalizations, we placed a speaker (FOXPRO; model INFERNO) in the natural vegetation 1–1.5 m above the ground. The researcher then retreated at least 10 m from the speaker, far enough to not disturb the responding birds but close enough to still visualize the speaker, before beginning playback of the stimuli.

To ensure residents were oriented to the experimental drum stimuli, each playback file began with two unmanipulated “whinny” calls. To avoid pseudo-replication, the whinny calls were selected from five different individuals and randomly assigned to precede the experimental drum stimuli. This initial orientation was necessary for our study because we wanted to measure differences in *how* residents engage (i.e., the correlated performance of defense behaviors), not just if they engaged at all. Previous work has shown the whinny call is regularly produced in both non-territorial and territorial contexts, suggesting that the whinny itself is not necessarily a territorial signal [84,88]. We therefore expect the impact of these pre-stimulus whinny calls on the residents’ territorial behavior to be minimal, and if there is an impact it would be evenly distributed across all treatment groups. With a standardized initial orientation to the playback site, we expected to see no difference in response latency between treatments because residents were already engaged with the simulated intruder before playback of the threatening experimental drum stimuli began. We used a Kruskall-Wallis test to compare response latency between drum treatments, confirming the success of this approach (paired defense: *χ*^*2*^_4_ = 5.0737, *p* = 0.2798; solo defense: *χ*^*2*^_4_ = 0.77291, *p* = 0.942). All statistical analyses were performed in RStudio (version 4.1.2) and the code used for all analyses is available in the supplemental materials (S1 Code).

After the introductory whinny calls, the 10-min experimental drum stimulus was broadcast to the resident(s) at a standard volume of 80 dB (measured 1 m from the speaker), as in previous studies [18,19]. The order of treatment stimuli was randomly determined at the beginning of the field season. During the experimental playback, a researcher verbally dictated observations of response behaviors into a recorder (iPhone X). Dictation was later transcribed into count values for each of the six response behaviors (Table 1), resulting in a dataset with total counts for how many times each behavior was performed during the 10-min defensive bout. Due to the experimental design, there was no way to blind the data collector to the playback treatments. To minimize the impact of this non-blind design, count data were not transcribed or analyzed until after field data collection had finished. Further, researchers transcribed the audio data twice and calculated an average percent error for behavior counts between these two transcriptions (average error = 1.01%). We used Kruskal-Wallis tests to determine if the number of times each behavior was performed during the 10-min stimulus varied based on threat context treatment (package: ‘stats’). Resident downy woodpeckers responded to playback either alone or as a pair. We used a Fisher’s exact test to determine if the number of defending residents (1 or 2) was a result of our experimental stimuli, followed by post-hoc pairwise Fisher’s exact tests to assess treatment differences (‘stats’). To assess the possibility that one sex was more likely to respond to the stimulus first we used a *χ*^*2*^ goodness of fit test (‘stats’).

**Table 1:** Ethogram summary of observed downy woodpecker response behaviors.

Behavior	Description	Citations
Whinny call (W)	A multi-note, melodic vocalization.	[84,88]
Pik call (P)	A single note vocalization. Also described as “chip” or “kick” call. Often produced repetitively.	[18,84,89]
Drum (D)	Rapidly hammering beak on a hard substrate to produce a percussive sound. Whole body gestural signal.	[32,84,89]
Wing flare (WF)	Broad spreading of wings in the direction of another bird. Often performed mid-flight. Also described as “full wing threat display.”	[89]
Crest raise (C)	Raising feathers of red cap to stand erect. Observed only in males. Measured in seconds.	[84,89]
Flyover (FO)	Physically flying from one side of the playback speaker to another.	[18]

In total, we conducted 86 simulated territorial intrusion sessions. We had to eliminate some of these sessions (N = 14) because we could not attribute each behavior performed during a simulated territorial intrusion to a specific resident bird. We also excluded sessions where non-resident birds responded to the playback if the focal residents engaged directly with the physical intruder (N = 9), thus making it impossible to determine which of the residents’ defense behaviors were in response to our experimental stimulus. This resulted in 63 simulated territorial intrusion sessions with usable behavioral data, with each playback drum treatment having at least 11 sessions (Average: N = 13; Long/Fast: N = 12; Long/Slow: N = 12; Short/Fast: N = 11; Short/Slow: N = 15; see S3 Table for further details).

### Manipulating threat context

To manipulate threat context, we created experimental drum stimuli by altering the length (# beats) and speed (beats/sec; Hz) of natural downy woodpecker drums (sourced from xeno-canto.org and Macaulay Library). Drum length and drum speed are both relevant signal components in territorial contests, with higher performance of either component eliciting a stronger defense response from resident birds. Previous studies have assessed these components in isolation, finding slightly different behavioral responses to each version of “high threat” [18,19]. With documented behavioral response differences, as well as known differences in the evolution of the length and speed components [90], we were interested in capturing defense in response to a broad range of threat types and therefore chose to manipulate both factors simultaneously. To accomplish this, we measured drums from local (Northeastern region of the USA) recordings of spontaneously drumming downy woodpeckers and calculated population averages (± SD) for drum length (17 beats ± 4.6) and speed (16.4 Hz ± 1.26). We created all stimuli for this study in Audacity (version 2.4.2) using five local drum recordings, each of which was engineered in five different ways so they would be represented in every treatment group. This approach allowed us to avoid any confounding effect of bird identity, since each individual was present in all five treatment groups. We randomly selected beats to replicate or remove from each existing drum to engineer drum stimuli of different lengths. To manipulate speed, we shortened each inter-beat interval by a consistent number of milliseconds such that the overall speed (beats/sec; Hz) met the target value. Length and speed were engineered to be 1.75 standard deviations from the mean (+1.75 SD for Long & Fast; −1.75 SD for Short & Slow) to represent biologically relevant “high” and “low” threat signal components. In total, we created five different stimuli treatments (quantitative details in S3 Table; for visual representation of drums see [34]). Based on previous studies in this system, the threats posed by Average, Long/Fast, and Short/Slow drum stimuli are hypothesized to be average threat, high threat, and low threat, respectively. We do not have a strong hypothesis for the perceived threat of Long/Slow or Short/Fast drum stimuli, therefore we refer to them as “mixed threat” throughout this study. To remain consistent with previous studies in this species, experimental drums were repeated on a 10-min audio track with 8 seconds of silence in between each drum [18,19].

### Quantifying territorial behavior networks

In downy woodpecker territorial defense, we found six unique behaviors that residents deploy during a simulated territorial intrusion (Table 1). We are interested in the patterns by which these behaviors are performed either alongside or in lieu of each other. To understand how response behaviors are or are not intertwined, we used correlational network analysis to quantify the relationships between each behavior’s deployment at the population level.

#### Generating a null expectation.

To determine if behaviors are deployed non-randomly throughout the population, we needed an expectation of random defense to which we could compare observed defense. Quantifying “random” defense involved three major steps: simulating behavior counts, simulating random simulated territorial intrusion responses, and bootstrapping correlation coefficients for these random defense bouts. We completed all three of these steps for each threat context separately such that the patterns observed in each context were tested against a context-specific null expectation. We chose to calculate null expectations in a threat context specific fashion because pooling the count data for a single null is a potentially inaccurate weighting of the different types of threats these birds face. We avoided pooling all threat contexts’ responses for a single null because we do not know how frequent each of the five treatments is in the natural population. So, including the counts from all treatments at an arbitrary weighting would be misinformed. By creating a null “random” expectation for each specific context, we eliminate this potential source of bias.

First, we simulated behavioral count data based on the actual occurrence of that behavior over the 10-min playback in each threat context. We began by fitting a frequency distribution to the observed counts (function: ‘fitdistr’, package: ‘MASS’) and compared AIC values to determine whether a negative binomial or Poisson distribution was the best fit for the data (S4 Table). Next, we simulated null counts (N = 10,000) based on the observed parameters from the best fit distribution (‘size’ and ‘mu’ for negative binomial; ‘lambda’ for Poisson).

Second, we used the null count distributions to simulate random behavioral responses. We began by creating a blank matrix where each row represented the simulated response to a single 10-min simulated territorial intrusion session. We filled rows with simulated behavior count values that were randomly sampled from our null count distributions (step 1). In this way, we simulated a data set of 10,000 simulated territorial intrusion sessions where the “performance” of each behavior was randomly selected and generated independently of all other behaviors. The result of this step is a defense simulation generated in the absence of any sort of behavior correlations, with behavioral occurrence based solely on the behavior’s observed performance frequency in that threat context.

Lastly, to generate a null expectation for the defense networks, we needed to calculate the null correlation coefficient values. To this end, we randomly sampled (with replacement) 100 simulations of simulated territorial intrusion sessions (matrix rows from step 2) and calculated Spearman’s rank correlation coefficients (rho) for each pair of behavior (function: ‘rcorr’, package: ‘Hmisc’). Importantly, we chose Spearman’s rank correlation because all of our behavioral data are nonparametric, requiring a rank-based approach [91]. We repeated the correlation calculation 10,000 times and input these data into a new matrix, creating a robust simulation of the expected correlations if all behaviors are performed at random and independent from each other. These null correlation values were used to calculate *p*-values for our observed data, thus creating a statistical test for whether the observed relationship (rho) between two behaviors is significantly more extreme than expected if all behaviors in that threat context were performed at random (further details below). One concern with nonparametric methods is the handling of ties, so we used a second nonparametric correlation coefficient that takes a different approach to tied values, Kendall’s tau, to confirm our results. We found that >97% of our correlations were the same between the two methods (S5 Table) and therefore chose to proceed with only Spearman’s rho for the full analysis.

#### Testing observed correlations for significance.

We tested for correlated performance of defense behaviors at the population level by comparing observed correlation values against the null distributions outlined above. If residents are intentionally organizing their behaviors into meaningful patterns, then actual behavior correlations will be more extreme than the null (random) expectation. To this end, we calculated Spearman’s rank correlation coefficients (rho) for observed behavioral responses to each treatment. Once we had the observed correlation coefficient for each behavior pair in each treatment, we tested for statistical significance by calculating the percentage of values in the matching null distribution which was more extreme than the observed experimental correlation value. Observed correlations were considered significant with an alpha cut off at 0.025 (for a two-tailed test). As in other uses of network analysis [41,92,93], we did not adjust for multiple testing because each correlation tested is a unique hypothesis.

All five threat contexts were met with enough paired defense to generate pair defense networks. For paired defense, we attributed each behavior to an individual sex resident and calculated correlations among the sex specific count values. This allowed us to characterize aspects of correlated behavior performance both within and between sexes. Notably, if there are significant positive correlations between female and male behaviors, that might indicate that at the population level residents are intentionally performing behaviors with their social mate. Two threat contexts received enough of a response from solo defenders to generate solo behavioral networks (Short/Fast, Short/Slow). In the case of solo defense, female and male responses followed the same behavioral patterns and were therefore combined into one, sex-inclusive pool of data for solo networks.

### Behavioral motifs

#### Quantifying motifs.

To quantify the structure underlying each population-level defense network, we applied spectral clustering to each correlation matrix to separate modular units of behavior (“motifs”). Spectral clustering is grounded in linear algebra and graph theory and is widely used across disciplines to partition network graphs into units based on their similarity and/or dissimilarity, as determined by the eigenvectors of the data’s Laplacian matrix [94]. This approach has traditionally been applied to unsigned (positively weighted) graphs, but we draw on a growing body of literature which outlines theory for spectral clustering of signed graphs [42,95,96]. Signed graphs have long been used to represent social dynamics and relationships [97–99]. Unlike unsigned graphs, which represent *both* indifference and antagonism with zero-weighted edges, signed graphs can represent likeness (positive-weight), indifference (zero-weight), and antagonism (negative-weight), although this poses a challenge for classical clustering algorithms. Spectral clustering is a state-of-the-art technique for clustering signed graphs, with many recently developed methods expanding on the approach of Kunegis et al. 2010 [42,100–102]. Not only does spectral clustering consider the directionality (sign) of each correlation, but it also considers the strength (weight) of the correlation such that weakly positively correlated behaviors are less likely to cluster than strongly positively correlated behaviors. Additionally, the magnitude of the eigenvalues reflects the dissimilarity between nodes in the graph, i.e., logical inconsistencies in the correlation signs of behavioral triads.

The signed Laplacian matrix ($\bar{L}$) has two components: *A* and $\bar{D}$. *A* is the adjacency matrix given by *A*_*ij*_ = *r*_*ij*_ where *r*_*ij*_ is the correlation coefficient (rho) between behaviors *i* and *j*, if the correlation coefficient is significant. $\bar{D}$ is the signed degree matrix for all behaviors, where the diagonal values are the sum of the absolute values of each corresponding row (i.e., $\bar{D_{ij}}=\sum_{all\;j}\left|r_{ij}\right|$) and the off-diagonal entries are zero. Finally, the signed Laplacian matrix $\bar{L}$ is given by $\bar{L}=\bar{D}-A$ As such, the signed Laplacian matrix $\bar{L}$ contains the degree of each behavioral node in the network as well as the correlation coefficients between significantly related behaviors.

Next, we performed *eigendecomposition* on each $\bar{L}$ using function ‘eigen’ (package: ‘base’) which yielded eigenvectors (EVs) and their associated eigenvalues. EVs were sorted by their corresponding eigenvalues from lowest eigenvalue to highest, such that EV1 corresponds to the lowest eigenvalue, EV2 the next lowest, and so on. If the adjacency matrix only contained positive correlation coefficients, then $\bar{L}$ would be the same as the unsigned Laplacian *L*, which would require throwing out the eigenvector associated with the smallest eigenvalue, which is redundant for clustering purposes [42]. However, in all $\bar{L}$ in our study, there was at least one negative correlation coefficient, so all eigenvectors were kept. To determine how many EVs are informative for clustering purposes, we used the eigengap heuristic [94,103]. To implement this heuristic, we plotted the eigenvalues to visualize the ‘eigengaps’ (difference between adjacent eigenvalues) and determine which eigengap shows the greatest departure from zero and/or the single largest eigengap (see supplement for eigengap plots; S1 Fig). By this approach, all EVs below this large eigengap are considered relevant for clustering purposes [104]. There is not one universally preferred method for determining the number of clusters in a network. However, the eigengap heuristic is sufficient for small graphs with distinct gaps between eigenvalues, particularly when negative correlations are frequent between clusters and infrequent within clusters, as in our study [104,105]. Importantly, in spectral clustering all relevant EVs are equally informative, and thus inform clustering in a non-hierarchical manner (as opposed to the hierarchal interpretation of PCA eigenvectors). Once relevant EVs are established, behaviors are separated based on whether they had a positive or negative value for each relevant EV. In this way, a relevant EV represents a “cut here” distinction at zero.

#### Defining motifs and related metrics.

We view motifs as the smallest meaningful unit of associated behaviors, as defined by considering a combination of spectral clustering EVs and the behavioral pair correlations. All motifs meet the following criteria: i) behavior(s) must fall on the same side of a relevant EV cut (i.e., eigenvalues for the relevant EV are all positive or all negative, see above); ii) if an EV cut indicates multiple behaviors are clustered, those behaviors must be connected by at least one positive correlation between them (that is, if a behavior is not significantly correlated or is only negatively correlated with other behaviors in the motif, then that behavior is not considered to be part of the motif in question); and finally iii) if a cluster is fully divided into smaller units of associated behaviors by a different relevant EV split such that the above criteria are met, then the motifs are determined to be the smaller (more split) clusters of behaviors. It is important to note that with these guidelines it is possible for a motif to be composed of a single behavior, but this should only occur when a behavior is mostly or completely connected to other nodes by negative correlations.

Behavioral motifs are useful for quantifying several aspects of territorial defense. First, the number of motifs present in a network is potentially a measure of the network’s modularity [106]. A larger number of motifs (or higher modularity) could indicate a more complex network [39,107]. Next, we calculate the number of motifs a single behavior appears in for a given threat context. The amount to which a given behavior can be rearranged to occupy different motifs may be indicative of how flexible the position of that behavior is within the broader population-level defense network. Finally, for paired defense networks, it may be informative to consider whether motifs include behaviors performed by one or both residents (between-sex motifs). Such within-motif correlations between female and male behaviors may be evidence of pair coordination [72,74,76,77].

#### Sequential behavioral analysis within motifs.

In addition to the correlative assessment of pair coordination, we quantified whether the behavior of one individual may directly influence the behavior of another through a sequential behavioral analysis. To this end, we tested the a priori positive behavior correlations present within motifs for evidence of direct, sequential transitions. This approach follows methods outlined by Green and Patek [92], and the full code used can be found in the supplementary material (S1 Code). For this analysis, we created a two-column dataset comprising all behavioral transitions that occurred in a given threat context. From these sequential data, we created context specific adjacency matrices where each value in the matrix represents the number of times a particular behavior was immediately followed by another behavior. Notably, it is possible and common for a single behavior to be repeated in sequence with itself.

To determine which of the observed sequential transitions occurred more frequently than expected by chance in the population, we used standard permutation methods found in sequential analyses [108]. First, we generated a null expectation for behavioral transitions by resampling the second column of the transition data 10,000 times, thus randomizing the sequence of behaviors while maintaining the context specific frequency of behavior performance. Each of these 10,000 iterations created an adjacency matrix, which we then used to generate a null distribution of the transition between two behaviors. From this null distribution, we calculated the 95% quantile for each possible transition. We then compared our observed adjacency matrix to the 95% quantile and determined which observed transitions were more frequent (greater than) the null expectation. These significant transitions were mapped onto the motifs, and any transitions that corresponded with a significant positive correlation between two behaviors within a motif is considered a meaningful behavioral transition for that threat context.

It is possible that the results of our transition analysis could be skewed by individual sessions. To quantify this potential bias, we tested a subset of our data (paired response to Average) with a leave one out approach, such that we calculated significant transitions between nodes with 11/12 sessions included. We repeated this calculation 12 times, removing each session once. When then compared these results to each of the significant transitions found when all 12 sessions are analyzed together and calculated an average error of 5%. Thus, we trust that the results of this analysis are statistically sound. However, see “Territorial defense is often a coordinated effort” in the Discussion for additional discussion of limitations.

## Supporting information

S1 Fig Eigenvalue plotting for applying the eigengap heuristic. All eigenvalues of the signed Laplacian matrix for a treatment are plotted on the y-axis. The associated eigenvector number is plotted on the x-axis. Red lines indicate the gap that was determined to be the most extreme departure from zero and/or the largest gap between two eigenvalues. The eigenvectors left of the red line were used to split networks into clustered motifs.
(PDF)

S1 Table All correlation coefficients for each threat context with the associated *p*-values. See Figs 2 and 4 for graphical illustration of significant relationships. *p < 0.025; **p < 0.01.
(PDF)

S2 Table Statistical comparison of behavior frequencies across threat contexts. (PDF)

S3 Table Summary of experimental drum stimuli length and speed for each of the five playback treatments. Sample size (territories) indicates the total number of territories receiving each treatment in the clean data set (63 sessions). Length and Speed indicate quantitative details of the drum stimuli for each treatment. N solo defense indicates the number of territorial responses that only involved one resident bird. N paired defense indicates the number of territorial responses that included both residents for a given territory. Shaded cells indicate that a given treatment and response type (solo/paired) met the minimum sample size to generate behavior networks (N = 5). For a visual representation of drum waveforms see Schuppe et al 2021 [34].
(PDF)

S4 Table Model fitting results to match frequency distributions to each of the response behaviors in each threat context. (PDF)

S5 Table Comparison of Spearman’s rho and Kendall’s tau significant correlations. Comparison of significant correlations for each defense network using two non-parametric correlation coefficients, Spearman’s rho and Kendall’s tau. Green cells indicate significant *p*-values.
(PDF)

S1 Data S1 Data_Moody et al 2025. All data necessary to recreate results.
(XLSX)

S1 Code S1 Code_Moody et al_Territorial Strategies. Code necessary to recreate analyses in R.
(R)

S1 Appendix README_Territorial Strategies. (RTF)
