# Supplementary material for: Social mates dynamically coordinate aggressive behavior to produce strategic territorial defense
Source: PLoS Comput Biol. 2025 Jan 24;21(1):e1012740. doi: 10.1371/journal.pcbi.1012740 (PMC11785317; doi:10.1371/journal.pcbi.1012740)

S1 Figure. Eigenvalue plotting for applying the eigengap heuristic.

All eigenvalues of the signed Laplacian matrix for a treatment are plotted on the y-axis. The associated eigenvector number is plotted on the x-axis. Red lines indicate the gap that was determined to be the most extreme departure from zero and/or the largest gap between two eigenvalues. The eigenvectors left of the red line were used to split networks into strategic tactics.

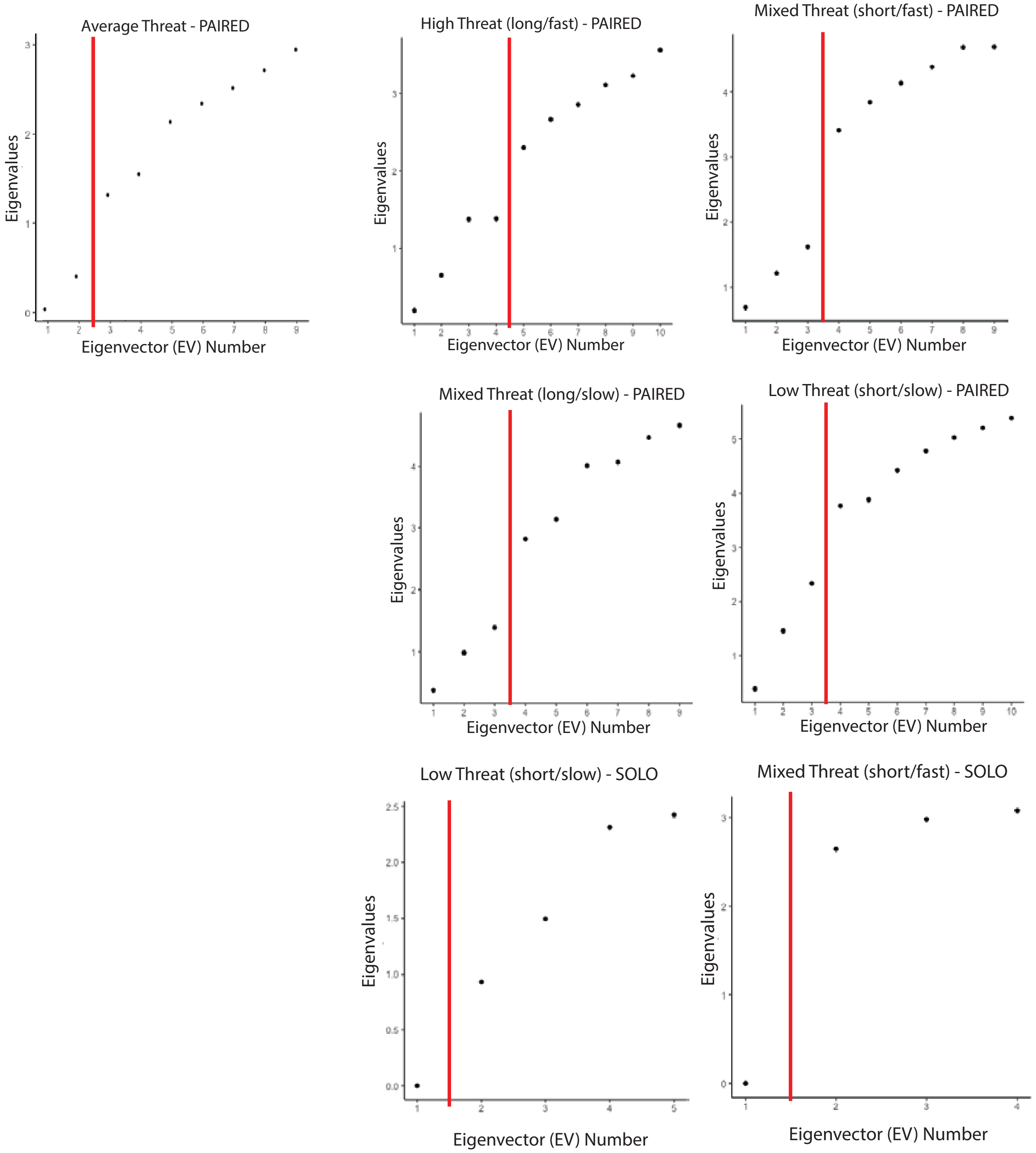

Supplement: S1 Fig — All eigenvalues of the signed Laplacian matrix for a treatment are plotted on the y-axis. The associated eigenvector number is plotted on the x-axis. Red lines indicate the gap that was determined to be the most extreme departure from zero and/or the largest gap between two eigenvalues. The eigenvectors left of the red line were used to split networks into clustered motifs. (PDF) [file pcbi.1012740.s001.pdf]
