# Supplementary material for: Social mates dynamically coordinate aggressive behavior to produce strategic territorial defense
Source: PLoS Comput Biol. 2025 Jan 24;21(1):e1012740. doi: 10.1371/journal.pcbi.1012740 (PMC11785317; doi:10.1371/journal.pcbi.1012740)
Supplement: S1 Table — See Figs 2 and 4 for graphical illustration of significant relationships. *p < 0.025; **p < 0.01. (PDF) [file pcbi.1012740.s002.pdf]

S1 Table. All correlation coefficients for each threat context with the associated *p*-values.

See Fig 2 and 4 for graphical illustration of significant relationships. *p <0.025; **p <0.01.

PAIRED DEFENSE

High Threat (Long/Fast)		Female					Male				
		Drum (D)	Whinny call (W)	Pik call (P)	Wing Flare (WF)	Flyover (FO)	Drum (D)	Whinny call (W)	Pik call (P)	Crest Raise (C)	Wing Flare (WF)
Female	D										
	W	NA									
	P	NA	0.579**								
	WF	NA	0.537**	0.177							
	FO	NA	0.0737	-0.161	0.239**						
Male	D	NA	0.322**	-0.0151	-0.214**	-0.107					
	W	NA	-0.400**	-0.670**	-0.321**	-0.234*	0.12				
	P	NA	0.244**	-0.153	0.412**	0.592**	-0.0151	0.122			
	C	NA	0.235**	0.479**	-0.364**	0.277**	-0.0234	-0.294**	-0.0386		
	WF	NA	-0.0597	0.412**	-0.111	0.418**	-0.214**	-0.321**	0.236**	0.546**	
	FO	NA	-0.0502	0.195*	-0.175	0.273**	-0.195	-0.00674	0.443**	0.653**	0.525**

PAIRED DEFENSE

Average Threat		Female					Male				
		Drum (D)	Whinny call (W)	Pik call (P)	Wing Flare (WF)	Flyover (FO)	Drum (D)	Whinny call (W)	Pik call (P)	Crest Raise (C)	Wing Flare (WF)
Female	D										
	W	NA									
	P	NA	-0.073								
	WF	NA	NA	NA							
	FO	NA	0.062	-0.434**	NA						
Male	D	NA	0.838**	-0.044	NA	0.179					
	W	NA	0.091	-0.474**	NA	0.281**	-0.123				
	P	NA	-0.134	-0.270**	NA	0.363**	-0.441**	0.596**			
	C	NA	0.19	0.572**	NA	-0.590**	0.0912	0.037	-0.444**		
	WF	NA	-0.281**	-0.263**	NA	0.397**	-0.208**	-0.172*	0.047	-0.280**	
	FO	NA	-0.404**	-0.172	NA	0.05	-0.388**	0.112	0.053	-0.117	0.354**

PAIRED DEFENSE

Low Threat short/slow		Female					Male				
		Drum (D)	Whinny call (W)	Pik call (P)	Wing Flare (WF)	Flyover (FO)	Drum (D)	Whinny call (W)	Pik call (P)	Crest Raise (C)	Wing Flare (WF)
Female	D										
	W	-0.338**									
	P	-0.515**	0.736**								
	WF	NA	NA	NA							
	FO	-0.424**	0.613**	0.112	NA						
Male	D	-0.255**	-0.147	0.449**	NA	-0.370**					
	W	-0.520**	-0.221*	0.174	NA	0.000	0.771**				
	P	-0.424**	-0.0613	0.112	NA	0.308**	0.601**	0.906**			
	C	0.228*	0.0989	0.0704	NA	-0.372**	-0.522**	-0.740**	-0.868**		
	WF	-0.441**	-0.362**	0.0973	NA	-0.240**	0.625**	0.884**	0.721**	-0.387**	
	FO	-0.624**	0.732**	0.927**	NA	0.321**	0.499**	0.426**	0.434**	-0.223**	0.295**

PAIRED DEFENSE

Mixed Threat (Long/Slow)		Female Drum (D)	Female Whinny call (W)	Female Pik call (P)	Female Wing Flare (WF)	Female Flyover (FO)	Male Drum (D)	Male Whinny call (W)	Male Pik call (P)	Male Crest Raise (C)	Male Wing Flare (WF)
Female	D										
	W	-0.304**									
	P	-0.722**	0.0472								
	WF	NA	NA	NA							
	FO	-0.754**	0.148	0.808**	NA						
Male	D	-0.281**	0.667**	0.252**	NA	0.347**					
	W	0.75**	-0.421**	-0.481**	NA	-0.503**	-0.188*				
	P	0.0834	-0.0133	-0.310**	NA	0.0183	-0.119	-0.358**			
	C	-0.368**	-0.378**	0.300**	NA	0.434**	-0.368**	-0.245**	-0.120		
	WF	NA	NA	NA	NA	NA	NA	NA	NA	NA	
	FO	-0.555**	0.394**	0.499**	NA	0.824**	0.289**	-0.555**	0.392**	0.247**	NA

PAIRED DEFENSE

Mixed Threat (Short/Fast)		Female Drum (D)	Female Whinny call (W)	Female Pik call (P)	Female Wing Flare (WF)	Female Flyover (FO)	Male Drum (D)	Male Whinny call (W)	Male Pik call (P)	Male Crest Raise (C)	Male Wing Flare (WF)
Female	D										
	W	-0.181									
	P	0.000	0.975**								
	WF	NA	NA	NA							
	FO	0.354**	-0.154	-0.2	NA						
Male	D	0.791**	0.287**	0.447**	NA	0.335**					
	W	-0.25**	-0.725**	-0.707**	NA	-0.353**	-0.395**				
	P	-0.25**	-0.725**	-0.707**	NA	-0.353**	-0.395**	1.0**			
	C	-0.25**	-0.181	-0.353**	NA	0.707**	-0.395**	-0.25**	-0.25**		
	WF	NA	NA	NA	NA	NA	NA	NA	NA	NA	
	FO	-0.725**	0.658**	0.564**	NA	-0.667**	-0.459**	-0.181	-0.181	-0.181	NA

SOLO DEFENSE

Mixed Threat Short/Fast	Drum (D)	Whinny call (W)	Pik call (P)	Crest Raise (C)	Wing Flare (WF)	Flyover (FO)
D						
W	-0.625**					
P	-0.647**	0.870**				
C	NA	NA	NA			
WF	NA	NA	NA	NA		
FO	0.806**	-0.688**	-0.718**	NA	NA	

SOLO DEFENSE

Low Threat Short/Slow	Drum (D)	Whinny call (W)	Pik call (P)	Crest Raise (C)	Wing Flare (WF)	Flyover (FO)
D						
W	0.607**					
P	0.264**	0.267**				
C	NA	NA	NA			
WF	-0.143	-0.607**	-0.351**	NA		
FO	0.439**	0.307**	0.736**	NA	-0.176	
