# Supplementary material for: Social mates dynamically coordinate aggressive behavior to produce strategic territorial defense
Source: PLoS Comput Biol. 2025 Jan 24;21(1):e1012740. doi: 10.1371/journal.pcbi.1012740 (PMC11785317; doi:10.1371/journal.pcbi.1012740)
Supplement: S2 Table — (PDF) [file pcbi.1012740.s003.pdf]

S2 Table. Statistical comparison of behavior frequencies across threat contexts.

					Pairwise Wilcoxon rank sum (adjusted *p* value, method=holm)									
**ID**	**behavior**	**Kruskal Wallis chi squared**	**df**	***p***	**avg vs long/fast**	**avg vs long/slow**	**avg vs short/fast**	**avg vs short/slow**	**long/fast vs long/slow**	**long/fast vs short/fast**	**long/fast vs short/slow**	**long/slow vs short/fast**	**long/slow vs short/slow**	**short/fast vs short/slow**
solo	drum	3.7262	4	0.44										
solo	whinny	4.3029	4	0.37										
solo	pik	3.4065	4	0.49										
solo	wing flare	1.5	4	0.83										
solo	flyover	6.0325	4	0.20										
male	drum	0.57837	4	0.97										
male	whinny	7.6023	4	0.11										
male	pik	2.5145	4	0.64										
male	crest	2.1357	4	0.71										
male	wing flare	11.807	4	0.02	1	1	1	0.39	1	1	0.53	--	0.13	0.45
male	flyover	9.8512	4	0.04	0.63	0.12	0.85	0.12	1	1	1	1	1	0.85
female	drum	4.764	4	0.31										
female	whinny	1.5895	4	0.81										
female	pik	1.7492	4	0.78										
female	wing flare	3.3	4	0.51										
female	flyover	5.4092	4	0.25										
