# Supplementary material for: Social mates dynamically coordinate aggressive behavior to produce strategic territorial defense
Source: PLoS Comput Biol. 2025 Jan 24;21(1):e1012740. doi: 10.1371/journal.pcbi.1012740 (PMC11785317; doi:10.1371/journal.pcbi.1012740)
Supplement: S3 Table — Sample size (territories) indicates the total number of territories receiving each treatment in the clean data set (63 sessions). Length and Speed indicate quantitative details of the drum stimuli for each treatment. N solo defense indicates the number of territorial responses that only involved one resident bird. N paired defense indicates the number of territorial responses that included both residents for a given territory. Shaded cells indicate that a given treatment and response type (solo/paired) met the minimum sample size to generate behavior networks (N = 5). For a visual representation of drum waveforms see Schuppe et al 2021 [34]. (PDF) [file pcbi.1012740.s004.pdf]

S3 Table. Summary of experimental drum stimuli length and speed for each of the five playback treatments.

Sample size (territories) indicates the total number of territories receiving each treatment in the clean data set (63 sessions). Length and Speed indicate quantitative details of the drum stimuli for each treatment. N solo defense indicates the number of territorial responses that only involved one resident bird. N paired defense indicates the number of territorial responses that included both residents for a given territory. Shaded cells indicate that a given treatment and response type (solo/paired) met the minimum sample size to generate behavior networks (N=5). For a visual representation of drum waveforms see Schuppe et al 2021.

Drum Treatment (length / speed)	Threat level	Sample Size (territories)	Length (# beats)	Speed (beats/sec; Hz)	N solo defense	N paired defense
Average	average	13	17	16.4	1	**12**
Long / Fast	high	12	25	18.6	2	**10**
Long / Slow	mixed	12	25	14.2	3	**9**
Short / Fast	mixed	11	9	18.6	**6**	**5**
Short / Slow	low	15	9	14.2	**8**	**7**
