# Supplementary material for: Social mates dynamically coordinate aggressive behavior to produce strategic territorial defense
Source: PLoS Comput Biol. 2025 Jan 24;21(1):e1012740. doi: 10.1371/journal.pcbi.1012740 (PMC11785317; doi:10.1371/journal.pcbi.1012740)
Supplement: S4 Table — (PDF) [file pcbi.1012740.s005.pdf]

S4 Table. Model fitting results to match frequency distributions to each of the response behaviors in each threat context.

Paired defense Model fitting for count distributions				
treatment	sex	behavior	negative binomial AIC	poisson AIC
long/fast	F	whinny	44.94	58.91
long/fast	F	pik	86.79	1644.50
long/fast	F	wing flare	10.61	8.61
long/fast	F	flyover	47.11	80.17
long/fast	M	drum	36.51	113.85
long/fast	M	whinny	28.67	27.93
long/fast	M	pik	60.53	101.84
long/fast	M	crest raise	58.45	806.09
long/fast	M	wing flare	16.83	33.29
long/fast	M	flyover	68.45	179.53
average	F	whinny	43.28	45.42
average	F	pik	126.31	2312.37
average	F	flyover	70.21	92.25
average	M	drum	47.30	150.27
average	M	whinny	26.77	33.12
average	M	pik	82.68	1032.77
average	M	crest raise	95.94	1831.09
average	M	wing flare	16.12	25.15
average	M	flyover	75.20	81.28
short/slow	F	drum	18.22	60.97
short/slow	F	whinny	21.94	20.82
short/slow	F	pik	55.73	144.09
short/slow	F	flyover	38.56	42.14
short/slow	M	drum	31.51	127.98
short/slow	M	whinny	28.56	27.61
short/slow	M	pik	62.96	619.00
short/slow	M	crest raise	20.09	18.14
short/slow	M	wing flare	18.77	16.75
short/slow	M	flyover	34.98	33.77
long/slow	F	drum	32.05	301.12
long/slow	F	whinny	39.14	42.75
long/slow	F	pik	91.63	2511.72
long/slow	F	flyover	44.66	48.56
long/slow	M	drum	24.24	58.41
long/slow	M	whinny	14.66	18.18
long/slow	M	pik	52.38	195.63
long/slow	M	crest raise	39.90	648.18
long/slow	M	flyover	51.23	59.31
short/fast	F	drum	14.77	21.57
short/fast	F	whinny	18.28	16.26
short/fast	F	pik	55.00	1034.40
short/fast	F	flyover	25.76	27.35
short/fast	M	drum	19.06	24.45
short/fast	M	whinny	12.04	11.05
short/fast	M	pik	20.97	204.30
short/fast	M	crest raise	24.65	984.87
short/fast	M	flyover	22.71	20.65

Solo defense Model fitting for count distributions			
treatment	behavior	negative binomial AIC	poisson AIC
short/slow	drum	18.69	68.94
short/slow	whinny	26.07	24.01
short/slow	pik	46.56	140.55
short/slow	wing flare	10.16	8.16
short/slow	flyover	35.64	37.76
short/fast	drum	32.58	65.16
short/fast	whinny	23.85	18.81
short/fast	pik	28.88	253.98
short/fast	flyover	30.52	29.91
