# Supplementary material for: Social mates dynamically coordinate aggressive behavior to produce strategic territorial defense
Source: PLoS Comput Biol. 2025 Jan 24;21(1):e1012740. doi: 10.1371/journal.pcbi.1012740 (PMC11785317; doi:10.1371/journal.pcbi.1012740)
Supplement: S5 Table — Comparison of significant correlations for each defense network using two non-parametric correlation coefficients, Spearman’s rho and Kendall’s tau. Green cells indicate significant p-values. (PDF) [file pcbi.1012740.s006.pdf]

S5 Table. Comparison of Spearman's rho and Kendall's tau significant correlations. Comparison of significant correlations for each defense network using two non-parametric correlation coefficients, Spearman's rho and Kendall's tau. Green cells indicate significant p-values.

Paired Defense -- Average Threat				
correlation	rho	pvalue rho	tau	pvalue tau
c.M-f.M	-0.1173788	0.094	-0.0365148	0.2703
c.M-wf.M	-0.2802596	5.00E-04	-0.2558409	1.00E-04
d.M-c.M	0.0912238	0.1918	0.09176629	0.1387
d.M-f.M	-0.3875342	0	-0.272255	0
d.M-p.M	-0.4405442	1.00E-04	-0.3900067	0
d.M-w.M	-0.1232437	0.1034	-0.120495	0.0834
d.M-wf.M	-0.2078565	0.0062	-0.1956464	0.0048
f.F-c.M	-0.5896246	0	-0.4707859	0
f.F-d.M	0.17870398	0.0387	0.10385162	0.0953
f.F-f.M	0.05008953	0.3249	-0.0165295	0.3974
f.F-p.M	0.36284589	2.00E-04	0.28971439	2.00E-04
f.F-w.M	0.28147147	0.003	0.23775852	0.0031
f.F-wf.M	0.39723067	0	0.34744115	0
p.F-c.M	0.5723223	0	0.44194174	0
p.F-d.M	-0.0438384	0.3397	-0.060833	0.2318
p.F-f.F	-0.4344081	0	-0.368106	0
p.F-f.M	-0.1722981	0.0443	-0.1936492	0.0031
p.F-p.M	-0.2702112	0.0047	-0.212132	0.0027
p.F-w.M	-0.473503	0	-0.3946026	0
p.F-wf.M	-0.2629489	0.0041	-0.2261335	0.0013
p.M-c.M	-0.444	1.00E-04	-0.38	0
p.M-f.M	0.05300977	0.3024	0.05477226	0.2346
p.M-wf.M	0.04670994	0.3665	0.04264014	0.3527
w.F-c.M	0.19038114	0.0253	0.14142136	0.0374
w.F-d.M	0.83826723	0	0.69523466	0
w.F-f.F	0.06248924	0.2788	0.09145491	0.1252
w.F-f.M	-0.4040618	0	-0.3504128	0
w.F-p.F	-0.073329	0.2527	-0.0714286	0.1878
w.F-p.M	-0.1342688	0.0733	-0.1010153	0.0843
w.F-w.M	0.09127043	0.1642	0.07958372	0.1602
w.F-wf.M	-0.2808218	0.0011	-0.2584383	6.00E-04
w.M-c.M	0.03692745	0.3649	0.02626129	0.3898
w.M-f.M	0.11185803	0.1135	0.0719195	0.1523
w.M-p.M	0.59576282	0	0.55148702	0
w.M-wf.M	-0.1724879	0.0219	-0.1679678	0.0162
wf.M-f.M	0.35372615	0	0.31139958	0

Paired Defense -- Long/Slow Threat				
correlation	rho	pvalue rho	tau	pvalue tau
c.M-f.M	0.23354968	0.0011	0.24713458	0.0038
d.F-c.M	-0.3464102	0	-0.3682427	0
d.F-d.M	-0.2666667	3.00E-04	-0.28125	4.00E-04
d.F-f.F	-0.6712486	0	-0.7542882	0
d.F-f.M	-0.4944132	0	-0.5547002	0
d.F-p.F	-0.6110101	0	-0.7219001	0
d.F-p.M	0.0942809	0.1221	0.08342798	0.1897
d.F-w.F	-0.2782433	3.00E-04	-0.3043904	7.00E-04
d.F-w.M	0.73029674	0	0.75	0
d.M-c.M	-0.3464102	0	-0.3682427	0
d.M-f.M	0.22473329	0.0032	0.28890635	0.0019
d.M-p.M	-0.0942809	0.1732	-0.1191828	0.155
d.M-w.M	-0.1825742	0.0083	-0.1875	0.0122
f.F-c.M	0.37370466	0	0.43370578	0
f.F-d.M	0.28767798	6.00E-04	0.34721203	4.00E-04
f.F-f.M	0.74348441	0	0.82352496	0
f.F-p.M	0.03390318	0.3709	0.01826503	0.4672
f.F-w.M	-0.4595725	0	-0.5028588	0
p.F-c.M	0.22677868	9.00E-04	0.30006002	6.00E-04
p.F-d.M	0.21821789	0.0036	0.25209209	0.0064
p.F-f.F	0.65915306	0	0.80779488	0
p.F-f.M	0.29424494	0	0.44916867	0
p.F-p.M	-0.2160247	0.0016	-0.3102834	2.00E-04
p.F-w.M	-0.41833	0	-0.4812667	0
p.M-c.M	-0.1224745	0.0673	-0.119636	0.1209
p.M-f.M	0.25425669	6.00E-04	0.39225703	1.00E-04
w.F-c.M	-0.3212877	1.00E-04	-0.3781026	1.00E-04
w.F-d.M	0.55648667	0	0.66731739	0
w.F-f.F	0.16675934	0.0193	0.14801927	0.0839
w.F-f.M	0.37518324	0	0.39397262	0
w.F-p.F	0	0.432	0.04722117	0.372
w.F-p.M	0	0.5752	-0.013395	0.5206
w.F-w.M	-0.3810004	0	-0.4214636	0
w.M-c.M	-0.2371708	3.00E-04	-0.2454951	0.0016
w.M-f.M	-0.492366	0	-0.5547002	0
w.M-p.M	-0.3227486	0	-0.3575485	0

Paired Defense -- Short/Fast Threat				
correlation	rho	pvalue rho	tau	pvalue tau
d.F-f.F	0.31622777	0	0.35355339	0
d.F-d.M	0.75592895	0	0.79056942	0
d.F-f.M	-0.6666667	0	-0.7254763	0
w.F-p.F	0.9486833	0	0.97467943	0
w.F-w.M	-0.6666667	0	-0.7254763	0
w.F-p.M	-0.6666667	0	-0.7254763	0
w.F-f.M	0.44444444	0	0.65789474	0
p.F-d.M	0.35856858	0	0.4472136	0
p.F-w.M	-0.6324555	0	-0.7071068	0
p.F-p.M	-0.6324555	0	-0.7071068	0
p.F-c.M	-0.3162278	0	-0.3535534	2.00E-04
p.F-f.M	0.31622777	0	0.56428809	0
f.F-d.M	0.35856858	0	0.3354102	4.00E-04
f.F-c.M	0.63245553	0	0.70710678	0
f.F-f.M	-0.5270463	0	-0.6668859	0
d.M-w.M	-0.3779645	0	-0.3952847	2.00E-04
d.M-p.M	-0.3779645	0	-0.3952847	0
d.M-c.M	-0.3779645	0	-0.3952847	0
d.M-f.M	-0.3779645	0	-0.4588315	0
w.M-p.M	1	0	1	0
p.M-c.M	-0.25	0	-0.25	0.0011
f.F-w.M	-0.3162278	1.00E-04	-0.3535534	0
f.F-p.M	-0.3162278	1.00E-04	-0.3535534	0
d.F-c.M	-0.25	5.00E-04	-0.25	0.0019
d.F-p.M	-0.25	6.00E-04	-0.25	0.0013
w.F-d.M	0.25197632	0.001	0.28676967	0.0018
d.F-w.M	-0.25	0.0014	-0.25	0.0034
w.M-c.M	-0.25	0.0025	-0.25	0.0038
p.F-f.F	-0.2	0.0047	-0.2	0.0365
w.M-f.M	-0.1666667	0.0198	-0.1813691	0.0263
p.M-f.M	-0.1666667	0.026	-0.1813691	0.0419
c.M-f.M	-0.1666667	0.0307	-0.1813691	0.0495
d.F-w.F	-0.1666667	0.0334	-0.1813691	0.0416
w.F-c.M	-0.1666667	0.0417	-0.1813691	0.0544
w.F-f.F	-0.1054093	0.1015	-0.1538968	0.0689
d.F-p.F	0	0.5248	0	0.5156

Paired Defense -- Long/Fast Threat				
correlation	rho	pvalue rho	tau	pvalue tau
c.M-f.M	0.65334352	0	0.55916142	0
c.M-wf.M	0.54590589	0	0.5	0
d.M-c.M	-0.023384	0.5015	-0.0340207	0.4426
d.M-f.M	-0.1950591	0.0341	-0.1556432	0.0308
d.M-p.M	-0.0151446	0.3646	-0.0318788	0.2785
d.M-w.M	0.12365484	0.151	0.11371471	0.1462
d.M-wf.M	-0.2141765	0.0077	-0.2041241	0.0047
f.F-c.M	0.27690969	0.0036	0.21629523	0.0039
f.F-d.M	-0.1073625	0.1567	-0.0993399	0.1348
f.F-f.M	0.27279296	0.0034	0.17316974	0.01
f.F-p.M	0.59181957	0	0.38002094	0
f.F-w.M	-0.2342878	0.0117	-0.1807426	0.0172
f.F-wf.M	0.41773368	0	0.37851665	0
p.F-c.M	0.47930042	0	0.3904344	0
p.F-d.M	-0.0151446	0.3515	-0.0318788	0.2613
p.F-f.F	-0.1614053	0.0548	-0.1013389	0.0963
p.F-f.M	0.19505485	0.0221	0.16671393	0.0075
p.F-p.M	-0.153125	0.0623	-0.1219512	0.0437
p.F-w.M	-0.6702076	0	-0.5800148	0
p.F-wf.F	0.1767767	0.0312	0.15617376	0.03
p.F-wf.M	0.41247896	0	0.36440544	0
p.M-c.M	-0.0386014	0.4042	-0.026029	0.4145
p.M-f.M	0.44274356	0	0.2857953	1.00E-04
p.M-wf.M	0.23570226	0.008	0.20823168	0.0051
w.F-c.M	0.2345588	0.007	0.18925832	0.0071
w.F-d.M	0.3220874	3.00E-04	0.23179316	0.0024
w.F-f.F	0.07371795	0.2378	0.05263158	0.2587
w.F-f.M	-0.0501688	0.3546	-0.0494771	0.2891
w.F-p.F	0.57916033	0	0.4306904	0
w.F-p.M	0.24369041	0.0069	0.22801257	9.00E-04
w.F-w.M	-0.3996675	0	-0.3614851	0
w.F-wf.F	0.53708616	0	0.48666426	0
w.F-wf.M	-0.0596762	0.2684	-0.0540738	0.2597
w.M-c.M	-0.294167	0.002	-0.2475938	0.0024
w.M-f.M	-0.0067412	0.4333	-0.0283183	0.3347
w.M-p.M	0.12247449	0.12	0.08700222	0.1538
w.M-wf.M	-0.3207501	0	-0.3094922	1.00E-04
wf.F-c.M	-0.3639373	0	-0.3333333	0
wf.F-d.M	-0.2141765	0.0037	-0.2041241	0.0031
wf.F-f.F	0.23870496	0.0069	0.21629523	0.0077
wf.F-f.M	-0.1751424	0.0392	-0.1524986	0.0344
wf.F-p.M	0.41247896	0	0.36440544	0
wf.F-w.M	-0.3207501	1.00E-04	-0.3094922	0
wf.F-wf.M	-0.1111111	0.085	-0.1111111	0.0743
wf.M-f.M	0.52542708	0	0.45749571	0

Paired Defense -- Short/Slow Threat				
correlation	rho	pvalue rho	tau	pvalue tau
c.M-f.M	-0.1839418	0.0206	-0.2231462	0.0144
c.M-wf.M	-0.3450328	2.00E-04	-0.3872983	0
d.F-c.M	0.21821789	0.0071	0.22771002	0.0116
d.F-d.M	-0.246183	4.00E-04	-0.2545875	0.0027
d.F-f.F	-0.3849002	0	-0.4236593	0
d.F-f.M	-0.5619515	0	-0.6236096	0
d.F-p.F	-0.4564355	0	-0.5149287	0
d.F-p.M	-0.3849002	0	-0.4236593	0
d.F-w.F	-0.3162278	2.00E-04	-0.337832	1.00E-04
d.F-w.M	-0.4682929	0	-0.5196746	0
d.F-wf.M	-0.421637	0	-0.4409586	0
d.M-c.M	-0.4834938	0	-0.5217492	0
d.M-f.M	0.41502868	0	0.49897013	0
d.M-p.M	0.49746834	0	0.60092521	0
d.M-w.M	0.62254302	0	0.77113566	0
d.M-wf.M	0.54494926	0	0.62546279	0
f.F-c.M	-0.3149704	0	-0.3721042	1.00E-04
f.F-d.M	-0.3553345	0	-0.3698001	0
f.F-f.M	0.21629523	0.0023	0.32081183	8.00E-04
f.F-p.M	0.22222222	9.00E-04	0.30769231	0.0015
f.F-w.M	0	0.5208	0	0.5182
f.F-wf.M	-0.1825742	0.0169	-0.2401922	0.006
p.F-c.M	0.05976143	0.2343	0.07035265	0.2498
p.F-d.M	0.33709993	0	0.44946658	0
p.F-f.F	0.15811388	0.018	0.11219364	0.1141
p.F-f.M	0.82078268	0	0.9266445	0
p.F-p.M	0.15811388	0.0161	0.11219364	0.1737
p.F-w.M	0.20519567	0.0029	0.17431926	0.0616
p.F-wf.M	0.11547005	0.1084	0.09731237	0.207
p.M-c.M	-0.7559289	0	-0.8682431	0
p.M-f.M	0.32444284	0	0.43403953	0
p.M-wf.M	0.66943868	0	0.72057669	0
w.F-c.M	0.06900656	0.237	0.09890707	0.1288
w.F-d.M	-0.1556998	0.0295	-0.147442	0.0675
w.F-f.F	0.54772256	0	0.61339562	0
w.F-f.M	0.59234888	0	0.73234727	0
w.F-p.F	0.6350853	0	0.73559957	0
w.F-p.M	-0.0608581	0.2053	-0.0613396	0.3273
w.F-w.M	-0.1777047	0.0174	-0.2207074	0.0181
w.F-wf.M	-0.2666667	7.00E-04	-0.361784	4.00E-04
w.M-c.M	-0.6131393	0	-0.7404398	0
w.M-f.M	0.36842105	0	0.42592593	0
w.M-p.M	0.81110711	0	0.90582163	0
w.M-wf.M	0.82928843	0	0.88388348	0
wf.M-f.M	0.23693955	0.0024	0.29462783	0.0013

Solo Defense -- Short/Fast Threat				
correlation	rho	pvalue rho	tau	pvalue tau
d-w	-0.6253054	0	-0.4803845	0
d-p	-0.6465791	0	-0.5773503	0
d-f	0.80645161	0	0.66666667	0
w-p	0.87038828	0	0.83205029	0
w-f	-0.6878359	0	-0.6405126	0
p-f	-0.7184212	0	-0.6735753	0

Solo Defense -- Short/Slow Threat				
correlation	rho	pvalue rho	tau	pvalue tau
d-w	0.60697698	0	0.56407607	0
d-f	0.43937478	0	0.40291148	0
w-wf	-0.606977	0	-0.5640761	0
p-wf	-0.3514998	0	-0.3223292	0
p-f	0.73648649	0	0.68181818	0
w-f	0.30669393	6.00E-04	0.22727273	0.0021
w-p	0.26669037	0.0036	0.22727273	0.0027
d-p	0.26362487	0.0043	0.24174689	0.0028
d-wf	-0.1428571	0.0481	-0.1428571	0.0428
wf-f	-0.1757499	0.0561	-0.1611646	0.0462
